# An extensive study on multiple ETL and HTL layers to design and simulation of high-performance lead-free CsSnCl_3_-based perovskite solar cells

**DOI:** 10.1038/s41598-023-28506-2

**Published:** 2023-02-13

**Authors:** M. Khalid Hossain, G. F. Ishraque Toki, Abdul Kuddus, M. H. K. Rubel, M. M. Hossain, H. Bencherif, Md. Ferdous Rahman, Md. Rasidul Islam, Muhammad Mushtaq

**Affiliations:** 1 Institute of Electronics, Atomic Energy Research Establishment, Bangladesh Atomic Energy Commission, Dhaka, 1349 Bangladesh; 2 College of Materials Science and Engineering, Donghua University, Shanghai, 201620 China; 3 Ritsumeikan Global Innovation Research Organization, Ritsumeikan University, Shiga, 525-0058 Japan; 4 Department of Materials Science and Engineering, University of Rajshahi, Rajshahi, 6205 Bangladesh; 5 Department of Physics, Chittagong University of Engineering and Technology, Chittagong, 4349 Bangladesh; 6 Higher National School of Renewable Energies, Environment and Sustainable Development, 05078 Batna, Algeria; 7 Department of Electrical and Electronic Engineering, Begum Rokeya University, Rangpur, 5400 Bangladesh; 8 Department of Electrical and Electronic Engineering, Bangamata Sheikh Fojilatunnesa Mujib Science & Technology University, Jamalpur, 2012 Bangladesh; 9 Department of Physics, University of Poonch Rawalakot, Rawalakot, 12350 Pakistan

**Keywords:** Solar cells, Solar cells, Electronic properties and materials, Structure of solids and liquids, Optical materials and structures

## Abstract

Cesium tin chloride (CsSnCl_3_) is a potential and competitive absorber material for lead-free perovskite solar cells (PSCs). The full potential of CsSnCl_3_ not yet been realized owing to the possible challenges of defect-free device fabrication, non-optimized alignment of the electron transport layer (ETL), hole transport layer (HTL), and the favorable device configuration. In this work, we proposed several CsSnCl_3_-based solar cell (SC) configurations using one dimensional solar cell capacitance simulator (SCAPS-1D) with different competent ETLs like indium–gallium–zinc–oxide (IGZO), tin-dioxide (SnO_2_), tungsten disulfide (WS_2_), ceric dioxide (CeO_2_), titanium dioxide (TiO_2_), zinc oxide (ZnO), C_60_, PCBM, and HTLs of cuprous oxide (Cu_2_O), cupric oxide (CuO), nickel oxide (NiO), vanadium oxide (V_2_O_5_), copper iodide (CuI), CuSCN, CuSbS_2_, Spiro MeOTAD, CBTS, CFTS, P3HT, PEDOT:PSS. Simulation results revealed that ZnO, TiO_2_, IGZO, WS_2_, PCBM, and C_60_ ETLs-based halide perovskites with ITO/ETLs/CsSnCl_3_/CBTS/Au heterostructure exhibited outstanding photoconversion efficiency retaining nearest photovoltaic parameters values among 96 different configurations. Further, for the six best-performing configurations, the effect of the CsSnCl_3_ absorber and ETL thickness, series and shunt resistance, working temperature, impact of capacitance, Mott–Schottky, generation and recombination rate, current–voltage properties, and quantum efficiency on performance were assessed. We found that ETLs like TiO_2_, ZnO, and IGZO, with CBTS HTL can act as outstanding materials for the fabrication of CsSnCl_3_-based high efficiency (*η* ≥ 22%) heterojunction SCs with ITO/ETL/CsSnCl_3_/CBTS/Au structure. The simulation results obtained by the SCAPS-1D for the best six CsSnCl_3_-perovskites SC configurations were compared by the wxAMPS (widget provided analysis of microelectronic and photonic structures) tool for further validation. Furthermore, the structural, optical and electronic properties along with electron charge density, and Fermi surface of the CsSnCl_3_ perovskite absorber layer were computed and analyzed using first-principle calculations based on density functional theory. Thus, this in-depth simulation paves a constructive research avenue to fabricate cost-effective, high-efficiency, and lead-free CsSnCl_3_ perovskite-based high-performance SCs for a lead-free green and pollution-free environment.

## Introduction

Industrial and academic societies have huge attention to the newly developed technology of lead (Pb) halide PSCs with their most noteworthy progress in the power conversion efficiencies (PCEs) exceeding 23%. The distinctive optoelectronic properties, simple solution-based synthesis technique, economical, and environmentally benign features have drawn scientific curiosity for all-inorganic metal halide perovskite nanocrystals of CsPbX_3_, (X = halogens) in recent years^1,2^. Although the performance of inorganic lead halide perovskites is excellent, the problematic issue of lead's inherent toxicity is yet to be addressed properly^3^. Therefore, CsSnX_3_ tin (Sn)-based perovskite has been an excellent option to be utilized in SCs, because of the non-toxicity of Sn^2+^ ions^4,5^. CsSnX_3_ perovskites transited from Sn^2+^ to more stable Sn^4+^ by oxidation, resulting in a high susceptibility to the surrounding environment^6^.

High symmetry of the perovskite structure with s^2^p^0^ electronic configuration of Sn, yields direct allowed transitions, high optical absorption coefficients, small carrier effective masses, and high defect tolerance, thereby resulting in superior optoelectronic performance^7–10^. The process of intrinsic ion migration in ABX_3_ (A = alkali metal or monovalent molecular cation; B = Pb or Sn; X = halogen) results in super device stability^11^. Due to these attractive features, these optoelectronic halide perovskite semiconductors have recently undergone several attempts of commercialization^12,13^.

Most solar cell devices use mesoporous and planar structures, which have a perovskite absorbing layer between the HTL and the ETL. The ETL is the layer through which electrons move from mesoscopic perovskite and the conventional nanoparticles of mesoporous metal oxides like TiO_2_^14–16^ and ZnO^17,18^, while holes are efficiently transported through a variety of HTLs as reported elsewhere^19–22^. The perovskite absorber is supported by these layers significantly, but the thickness, carrier concentration, and associated bulk defects need to be adjusted to obtain the best cell performance with superior stability. Several semiconductor materials like TiO_2_, ZnO, WO_3_, and SnO_2_^23^ have been used as ETL, whereas TiO_2_ with an anatase structure is found to be a promising one with a wide band gap and lower mid-gap defect states, along with high electronic mobility^24–26^. ETLs such as TiO_2_ including several semiconductors including ZnO, SnO_2_ have rarely been studied yet, and therefore an extensive investigation on the usage of promising multiple semiconductors such as ETLs for exploring the full potential of CsSnX_3_ perovskite-based solar cells is required^27^.

Furthermore, the HTLs affect solar cell performance, durability, and manufacturing cost significantly^28–30^. Conventionally, inorganic/organic small molecule and polymeric HTLs may be categorized depending on chemical structure and content. Inorganic HTLs (e.g., CuI, CuSCN, NiO)^31^ are more chemically stable and inexpensive^32^ in comparison to organic HTLs like Spiro-MeOTAD, PEDOT: PSS, and PTAA^33–35^. But the poor carrier extraction hindered obtaining high-performance photovoltaics performance, while the small molecule-based HTLs are too unstable but help to achieve comparable PCEs^36^. Polymeric HTLs like Spiro MeOTAD and P3HT^31^ are more stable at high temperatures, water resistant, and compatible with other materials. However, copper barium thiostannate (CBTS) is an earth-abundant, air-stable thin-film material with an adjustable bandgap, high absorption coefficient, non-centrosymmetric crystal structure, and large atomic size^37,38^. Thus, CBTS is a potential and competent material to be used as HLT for designing high-performance SCs.

For better prediction of the suitability of the title compound in PV applications, first-principle calculations using CASTEP software were also used to assess the structural, electronic, and optical characteristics of the CsSnCl_3_ absorber within the context of density functional theory (DFT). Some experimental and theoretical reports on this compound have been found in recent times^39,40^. The title perovskite has been synthesized by the low-temperature hot-injection technique with a temperature of 200 °C coupled with a theoretical study based on DFT. The electronic and optical properties were calculated using GGA + U potential and consistency between experimental and theoretical results was found^41^. Islam et al.^42,43^ have studied the optoelectronic, structural, and mechanical properties of CsSnCl_3_ compound under the application of hydrostatic pressure and metal (Cr/Mn)-doping in CsSnCl_3_ perovskites using the first principles method based on DFT. It was reported that the electronic band gap was significantly decreased with the effect of pressure and metal doping. Some other authors^44–47^ have reported the interesting physical properties of some halide perovskite materials for optoelectronic and photovoltaic (PV) applications. Here, we have revisited the structural, electronic, and optical properties of CsSnCl_3_ materials to provide additional new information. Some minor changes in properties may sometimes significantly impact the device's performance.

In this article, we have extensively investigated different ETLs and HTLs to discover the best possible combination for the CsSnCl_3_ absorber layer theoretically choosing 96 configurations using SCAPS-1D^48,49^. To minimize the time consumption and cost required for fabricating experimentally a such huge number of SC configurations, we conducted a numerical analysis to obtain a highly efficient SC architecture. From such perspectives, the CsSnCl_3_ absorber-based SCs with a wide variety of ETLs such as PCBM, TiO_2_, ZnO, C_60_, IGZO, SnO_2_, WS_2_, CeO_2,_ and HTLs like Cu_2_O, CuSCN, CuSbS_2_, NiO, P3HT, PEDOT: PSS, Spiro MeOTAD, CuI, CuO, V_2_O_5_, CBTS, and CFTS for 96 different combinations using ITO/ETLs/CsSnCl_3_/HTLs/Au structure has been studied. After obtaining the most promising configurations from 96 heterostructures, we further examined the impact of the CsSnCl_3_ absorber and ETL thickness on PV performance, series and shunt resistance, and the working temperature of the best-performing six devices. Furthermore, the effect of capacitance, Mott-Schottky, generation and recombination rate, *J–V* characteristics, and quantum efficiency were evaluated. The six best structures were further validated by the wxAMPS simulation. Finally, a comparative study of obtained SC parameters with recent reports has been studied. Thus, several promising and competitive configurations for CsSnCl_3_-based high-efficiency SC have been proposed, which provide a constructive research avenue for designing and fabricating cost-effective, high-efficiency, and lead-free CsSnCl_3_-based SCs.

## Materials and methods

### First principle calculations of the CsSnCl_3_ absorber layer

In the framework of DFT, the presented first principle calculations are performed employing the CASTEP program^50,51^. The used basic set of the valence electronic structure for Cs, Sn, and Cl are 5*s*^2^5*p*^6^6*s*^1^, 4*d*^10^5*s*^2^5*p*^2^, and 2*p*^6^3*s*^2^3*p*^5^, respectively. The experimentally obtained structural data are listed in Table 1**,** which was used to build the crystal structure of CsSnCl_3_ perovskite. In this cubic unit cell, Cl atoms occupy the Wyckoff location of 3c (0.0, 0.5, 0.5); while Sn and Cs elements are located at the Wyckoff positions of 1b (0.5, 0.5, 0.5) and 1a (0.0, 0.0, 0.0), respectively. Herein, ultrasoft pseudopotential rituality of the Vanderbilt type^52^ was set to model the interactions between valence electrons and ion ores, and the generalized gradient approximation (GGA) was used as the exchange–correlation potential. We optimized the cubic phase's structure with *Pm*3̅*m* symmetry since the choice of exchange–correlation functionals (XCs) is an essential factor in DFT computations. Utilizing various XCs, the Broyden-Fletcher-Goldfarb-Shannon (BFGS) algorithm^52^ was used to determine the least energy state of the entire stable structure. A comparison of the formation energy (Δ*E*_f_, eV/atom) of the optimized structure and the predicted lattice constants with the given experimental data was done. The best-produced data by XC was used to compute all the properties of CsSnCl_3_ perovskite. The wave function’s cutoff energy was adjusted to 520 eV for the computer simulation of the CsSnCl_3_ solar absorber. The irreducible Brillouin zone was modeled using a 16 × 16 × 16 (*k*-point) Monkhorst–Pack grid^53^. Nonetheless, to view the Fermi surface topology and electronic charge density map, a larger size of the *k*-point mesh, 19 × 19 × 19 was employed. In such calculations, 0.03 eV/Å was the highest atom potency, 0.001 Å was the utmost atom, and the maximum stress was set as 0.05 GPa. Total energy 1 × 10^−5^ eV/atom was utilized for the converging tolerances of geometry optimization.

**Table 1:** Crystallographic data of CsSnCl_3_ absorber material.

Lattice parameters (Å)			axial angles (°)			Lattice type	Unit-cell volume (Å^3^)	Ref.	
a	*b*	c	*α*	*β*	*γ*				
5.62488	5.62488	5.62488	90	90	90	P; S.G. Pm$\overline{3}$m (#221)	177.967336	^42,54^	
5.629	5.629	5.629	90	90	90	P; S.G. Pm$\overline{3}$m (#221)	178.38	This study	

Structural parameters									
Elements		x	y	z	Occupancies		Sites	Symmetry	Ref
1	Cs/Cs0	0.0	0.0	0.0	1.0	1.0	1a	m − 3 m	^54^
2	Sn/Sn1	0.5	0.5	0.5	1.0	1.0	1b	m − 3 m	^54^
3	Cl/Cl2	0.0	0.0	0.0	1.0	1.0	3c	4/mmm	^54^

### SCAPS-1D numerical simulation

Modeling and simulation made it much simpler to understand the foundations and function of SCs, and it reveals the primary aspects that have the utmost impact on device performance. The behavior of semiconductor materials, when they are in a stable condition, may be solved numerically using the SCAPS-1D software’s numerical solution solving the one-dimensional Poisson and carrier continuity Equations^55^. The Poisson’s equation, which connects electric field (E) across a p–n junction with the space charge density, is as stated in Eq. (1):

1 $$\frac{d}{dx}\left. {\left( { - \varepsilon \left( x \right)\frac{d\psi }{{dx}}} \right.} \right) = q\left[ {p\left( x \right) - n\left( x \right) + N_{d}^{ + } \left( x \right) - N_{a}^{ - } \left( x \right)} \right]$$

In this case, n/p denotes the total electron/hole density, ${N}_{d}^{+}/{N}_{a }^{-}$ denote the ionized donor/acceptor concentration. Additionally, ε is the permittivity of the medium, q is the electronic charge, and ψ denotes the electrostatic potential. Equations (2) and (3) are considered to constitute the continuity of the electrons and holes, respectively, as shown in the following equations:

2 $$\frac{{\partial j_{n} }}{\partial x} = q\left( {R_{n} - G + \frac{\partial n}{{\partial t}}} \right)$$

3 $$\frac{{\partial j_{p} }}{\partial x} = - q\left( {R_{p} - G + \frac{\partial p}{{\partial t}}} \right)$$

In this equation, ${j}_{n}/{j}_{p}$ represent the electron/hole density, ${R}_{n}/{R}_{p}$ represent the electron/hole net recombination rates per unit volume, and $G$ is the generation rate per unit volume.

Again, to compute the absorption data for every layer, the new E_g_-sqrt system was used. This system is the updated version of the prior SCAPS model, which was the conventional sqrt $(h\vartheta -Eg)$ law model. These guidelines may be found in something called the "Tauc laws." The updated Eg-sqrt model may be characterized as follows in Eq. (4):

4 $$\alpha \left( {h\upsilon } \right) = \left( {\alpha_{0} + \beta_{0} \frac{{E_{g} }}{h\upsilon }} \right)\sqrt {\frac{h\upsilon }{{E_{g} }} - 1}$$

In this context, α stands for the optical absorption constant, $h\upsilon$ is photon energy, and E_g_ is the bandgap. The following Eqs. (5) and (6) show how the model constants α_0_ and β_0_ are connected to the traditional model constants A and B. The dimension of the absorption constant (for example, cm^−1^) is used for both of these model constants.

5 $$\alpha_{0} = A\sqrt {E_{g} }$$

6 $$\beta_{0} = \frac{B}{{\sqrt {E_{g} } }}$$

### wxAMPS numerical simulation

Poisson’s equation in a one-dimensional space is represented by Eq. (7) in the wxAMPS numerical simulation^56^. The electrostatic potential $\psi^{\prime}$, and the concentrations of ionized donor denoted by ${N}_{D}^{+}$ and ionized acceptor denoted by ${N}_{A}^{-}$, as well as the free electron denoted by n, and the free hole denoted by p, the trapped electron denoted by nt, and the trapped hole denoted by pt and the variable is x. The free electrons that exist in the delocalized states of the conduction band can be described by the Eq. (8) ^56^.

7 $$\frac{d}{dx}\left( { - \varepsilon \left( x \right)\frac{{d\psi^{\prime}}}{dx}} \right) = q.\left[ {p\left( x \right) - n\left( x \right) + N_{D}^{ + } \left( x \right) - N_{A}^{ - } \left( x \right) + pt\left( x \right) - nt\left( x \right)} \right]$$

8 $$\frac{1}{q}\left( {\frac{dJn}{{dx}}} \right) = - G_{op} \left( x \right) + Rx$$

where the electrostatic potential is denoted by $\psi^{\prime}$, and nt/pt denotes the trapped electron/hole density. Similarly, the continuity equation for the free holes in the delocalized states of the valence band takes the form Eq. (9).

9 $$\frac{1}{q}\left( {\frac{dJp}{{dx}}} \right) = G_{op} \left( x \right) {-} Rx$$

here the electron current density is Jn, and the hole current density is Jp. The net recombination rate that occurs as a consequence of band-to-band (direct) recombination and SRH (indirect) recombination that occurs through gap states are taken care of by the term R(x). Equation (10) represents the rate of net direct recombination ^56^.

10 $$R_{D} \left( x \right) \, = \beta \left( {np \, - \, ni^{2} } \right)$$

In this case, n and p refer to the band carrier concentrations that are present in the devices after they have been subjected to a volt bias, a light bias, or even both of these types of biases. In addition, the material bandgaps under investigation determine the proportionality constant (β). In the continuous equation, the word “G_op_(x)” stands for the optical generation rate as a function of x due to externally supplied light, which is a part of the optical generation rate.

### CsSnCl_3_ perovskite SC structure

Simulations of perovskite (CsSnCl_3_) absorber-based SCs were carried which consist of an ETL defined as the n region, the perovskite layer is the p-region as it is doped p-type, and the HTL also constitutes the p region. When the cell is illuminated by light, excitons (the constituent parts of a restricted state) are dominantly generated in the perovskite layer. The photogenerated carriers of holes (electrons) with longer diffusion lengths allow them to enter the p (n) region. At the boundary between the ETL and perovskite, the generated excitons (h-e pairs) are dissociated and the electrons are transported through the ETL to the respective electrode, while the holes travel efficiently through the HTL. The existence of the built-in field in the space between the ETL or HTL and the perovskite interface drives the excitons dissociation and their transportation, which accelerates electron and hole movement to respective contacts.

**Figure 1:**
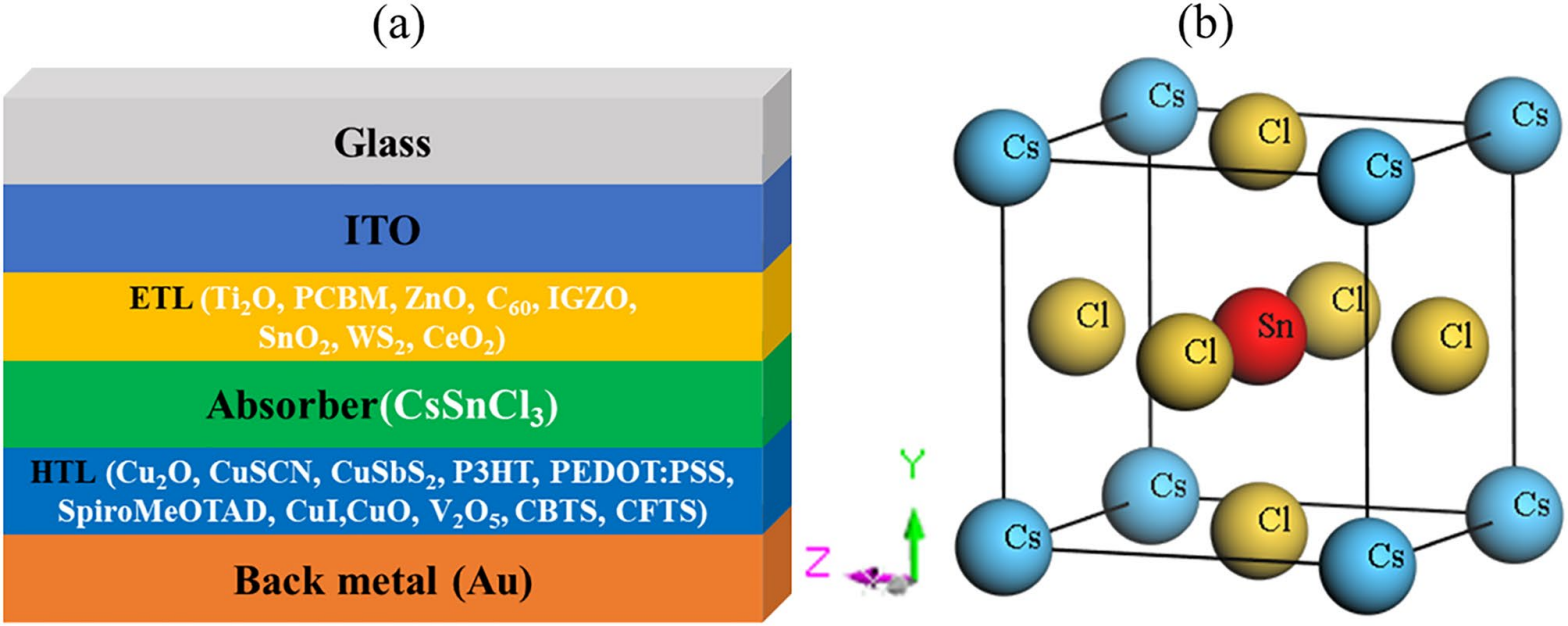
(**a**) Design configuration of the CsSnCl_3_-based PSC, and (**b**) the optimized crystal structure of CsSnCl_3_ perovskite.

Tables 2 and 3 show the list of simulation parameters of the TCO, ETL, absorber, and HTL layers. Herein, SC architecture comprises indium-doped tin oxide (ITO) as the front contact, 8 ETLs, specified CsSnCl_3_ perovskite as an absorber, and 12 HTLs with gold (Au) as the back contact metal (Fig. 1a). Furthermore, the simulation parameters of interface defect density are outlined in Table 4. The simulation is run under the conditions of an AM1.5G simulated solar light exposure with a power density of 100 mW/cm^2^ at the ambient temperature (273 K). The only exception to this is the evaluation of the influence of working temperature on the efficiency of the device. During the initial simulation and further optimization, a standard absorption model with sqrt $(h\vartheta -Eg)$ law (SCAPS traditional) was used.

**Table 2:** Input optimization parameters of TCO, ETL, and absorber layer of the study ^56,57^.

Parameters	ITO	TiO_2_	PCBM	ZnO	C_60_	IGZO	SnO_2_	WS_2_	CeO_2_	CsSnCl_3_
Thickness (nm)	500	30	50	50	50	30	100	100	100	800*
Band gap, E_g (eV_	3.5	3.2	2	3.3	1.7	3.05	3.6	1.8	3.5	1.52
Electron affinity, X (eV)	4	4	3.9	4	3.9	4.16	4	3.95	4.6	3.90
Dielectric permittivity (relative), ε_r_	9	9	3.9	9	4.2	10	9	13.6	9	29.4
CB effective density of states, N_C_ (1/cm^3^)	2.2 × 10^18^	2 × 10^18^	2.5 × 10^21^	3.7 × 10^18^	8.0 × 10^19^	5 × 10^18^	2.2 × 10^18^	1 × 10^18^	1 × 10^20^	1 × 10^19^
VB effective density of states, N_V_ (1/cm^3^)	1.8 × 10^19^	1.8 × 10^19^	2.5 × 10^21^	1.8 × 10^19^	8.0 × 10^19^	5 × 10^18^	1.8 × 10^19^	2.4 × 10^19^	2 × 10^21^	1 × 10^19^
Electron mobility, µ_n_ (cm^2^/Vs)	20	20	0.2	100	8.0 × 10^−2^	15	100	100	100	2
Hole mobility, µ_h_ (cm^2^/Vs)	10	10	0.2	25	3.5 × 10^−3^	0.1	25	100	25	2
Shallow uniform acceptor density, N_A_ (1/cm^3^)	0	0	0	0	0	0	0	0	0	1 × 10^15^*
Shallow uniform donor density, N_D_ (1/cm^3^)	1 × 10^21^	9 × 10^16^	2.93 × 10^17^	1 × 10^18^	1 × 10^17^	1 × 10^17^	1 × 10^17^	1 × 10^18^	10^21^	0*
Defect density, N_t_ (1/cm^3^)	1 × 10^15^*	1 × 10^15^*	1 × 10^15^*	1 × 10^15^*	1 × 10^15^*	1 × 10^15^*	1 × 10^15^*	1 × 10^15^*	1 × 10^15^*	1 × 10^15^*

*This study.

**Table 3:** Input optimization parameters HTL the study^56^.

HTL	Cu_2_O	CuSCN	CuSbS_2_	P3HT	PEDOT: PSS	Spiro-MeOTAD	NiO	CuI	CuO	V_2_O_5_	CFTS	CBTS
Thickness (nm)	50	50	50	50	50	200	100	100	50	100	100	100
Band gap, Eg (eV)	2.2	3.6	1.58	1.7	1.6	3	3.8	3.1	1.51	2.20	1.3	1.9
Electron affinity, Χ (eV)	3.4	1.7	4.2	3.5	3.4	2.2	1.46	2.1	4.07	4.00	3.3	3.6
Dielectric permittivity (relative), εr	7.5	10	14.6	3	3	3	10.7	6.5	18.1	10.00	9	5.4
CB effective density of states, NC (1/cm^3^)	2 × 10^19^	2.2 × 10^19^	2 × 10^18^	2 × 10^21^	2.2 × 10^18^	2.2 × 10^18^	2.8 × 10^19^	2.8 × 10^19^	2.2 × 10^19^	9.2 × 10^17^	2.2 × 10^18^	2.2 × 10^18^
VB effective density of states, NV (1/cm^3^)	1 × 10^19^	1.8 × 10^18^	1 × 10^1^	2 × 10^21^	1.8 × 10^19^	1.8 × 10^19^	1 × 10^19^	1 × 10^19^	5.5 × 10^20^	5.0 × 10^18^	1.8 × 10^19^	1.8 × 10^19^
Electron mobility, µn (cm^2^/Vs)	200	100	49	1.8 × 10^−3^	4.5 × 10^–2^	2.1 × 10^–3^	12	100	100	3.2 × 10^2^	21.98	30
Hole mobility, µh (cm^2^/Vs)	8600	25	49	1.86 × 10^−2^	4.5 × 10^–2^	2.16 × 10^–3^	2.8	43.9	0.1	4.0 × 10^1^	21.98	10
Shallow uniform acceptor density, N_A_ (1/cm^3^)	1 × 10^18^	1 × 10^18^	1 × 10^18^	1 × 10^18^	1 × 10^18^	1.0 × 10^18^	1 × 10^18^	1.0 × 10^18^	1 × 10^18^	1 × 10^18^	1 × 10^18^	1 × 10^18^
Shallow uniform donor density, N_D_ (1/cm^3^)	0	0	0	0	0	0	0	0	0	0	0	0
Defect density, N_t_ (1/cm^3^)	1.0 × 10^15^*	1 × 10^15^*	1 × 10^15^*	1 × 10^15^*	1 × 10^15^*	1.0 × 10^15^*	1 × 10^15^*	1.0 × 10^15^*	1 × 10^15^*	1 × 10^15^*	1 × 10^15^*	1 × 10^15^*

*In this study.

**Table 4:** Input parameters of interface defect layers^58^.

Interface	Defect type	Capture cross section: Electrons/holes (cm^2^)	Energetic distribution	Reference for defect energy level	Total density (cm^−3^) (integrated over all energies)
ETL/CsSnCl_3_	Neutral	1.0 × 10^−17^ 1.0 × 10^−18^	Single	Above the VB maximum	1.0 × 10^10^
CsSnCl_3_/HTL	Neutral	1.0 × 10^−18^ 1.0 × 10^−19^	Single	Above the VB maximum	1.0 × 10^10^

## Results and discussions

### Analysis of DFT results

#### Structural properties of CsSnCl_3_compound

The structural and phase stability of any material is a crucial criterion for the absorption of solar energy and applications in photovoltaic cells. The compound CsSnCl_3_ belongs to the cubic crystal family with the space group Pm$\overline{3 }$m (No. 221). The crystallographic data of our studied SC absorber material is presented in Table 1. The Cs atom is at the eight corners, Cl atom is at the six faces, and the Sn atom is in the body-centered position with an SnCl_6_ octahedral structure in the unit cell of CsSnCl_3_ perovskite. Figure 1b illustrates the crystal structure of CsSnCl_3_ perovskite. The title compound contains five atoms in a unit cell, meaning that the unit cell has one formula unit. The crystal structure is optimized using the BFGS optimization technique to obtain minimum volume and energy data^59^. The estimated lattice parameters *a* = *b* = *c* = 5.629 Å and volume = 178.38 Å^3^ for the title perovskite compounds agree well with other experimental and theoretical data^41,42^ as shown in Table 1. The physical properties studied are expected to be reliable since the unit cell dimension mismatch is less than 1.0%. Furthermore, the optimized structure’s extremely low and negative formation energy value (ΔE_f_ = − 1876.16 eV/atom) demonstrates its structural stability, which is compatible with solar system assembly.

#### Electronic band structure (BS) and density of states (DOS) of CsSnCl_3_ compound

**Figure 2:**
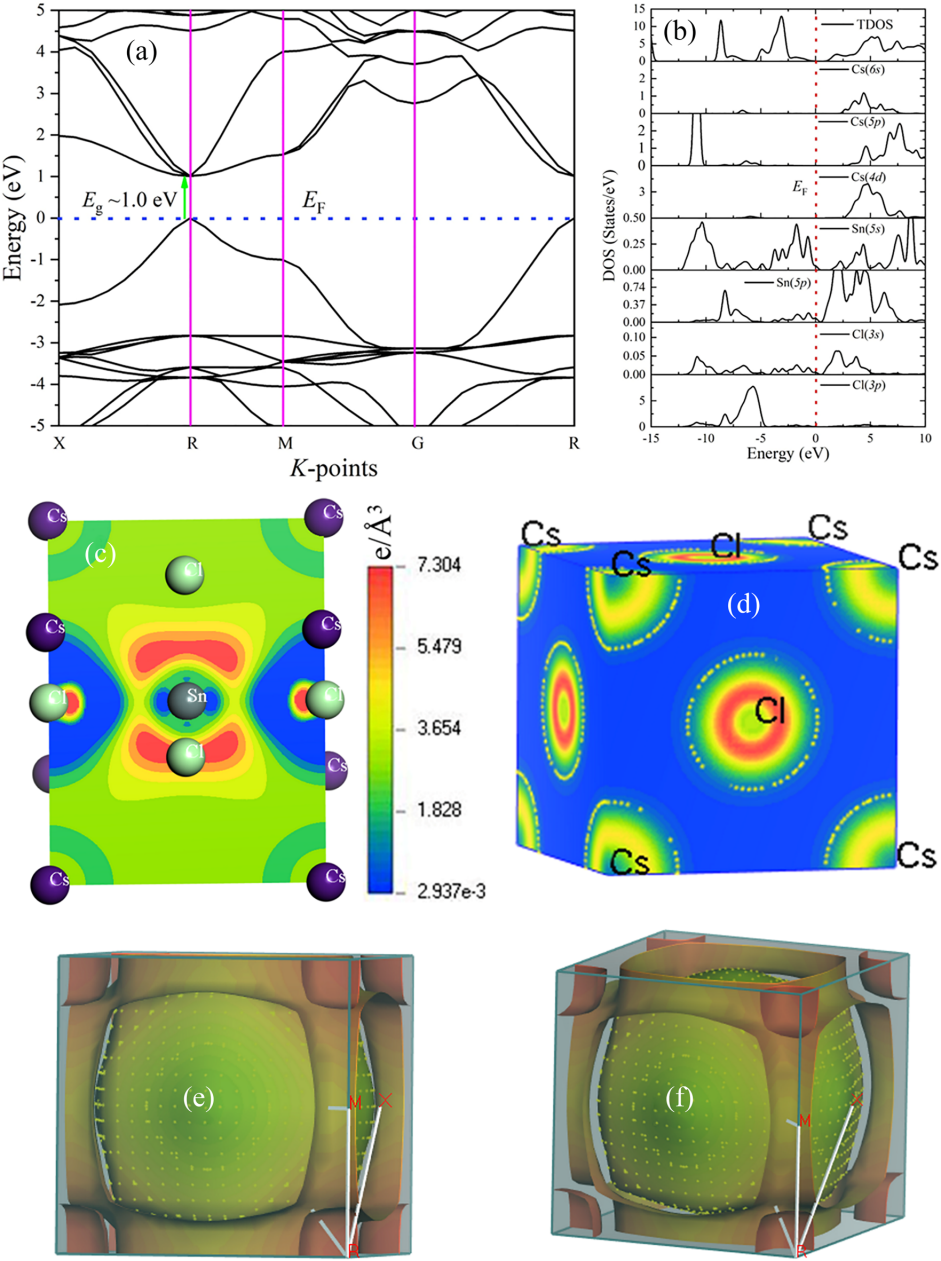
The electronic (**a**) band structure and (**b**) DOS of halide perovskite, CsSnCl_3_, (**c**) and (**d**) The mapping images of electron density difference in the (110) and (001) planes for halide perovskite, CsSnCl_3_, (**e**) and (**f**)The Fermi surface topology of CsSnCl_3_ perovskite along (001) plane of two different orientations in the same Brillouin zone direction.

The electronic properties of CsSnCl_3_ halide lead-free Perovskite, are studied using a GGA within the framework of DFT as implemented in the CASTEP code^51,60^. The computed electronic properties such as band structure (BS) and density of states (DOS) are calculated along the highly symmetric X–R–M–G–R direction of the first Brillouin zone and are depicted in Fig. 2a,b, respectively. In Fig. 2a, the horizontal dotted line indicates the Fermi level (*E*_F_) and is set to zero (0). The energy band gap defined by the difference between the conduction and valence bands is estimated to be ~ 1.0 eV using the GGA functional. The calculated band gap (*E*_g_) value is well justified with other reports using GGA/LDA functional^42^, but this value is much lower than some reported band gap values using non-local functional^41,57^. This underestimation of the band gap, especially for semiconductor materials using local functions like GGA and LDA, are widespread^61,62^. The strong Coulomb correlation and electron–electron interaction of the material might affect the band gap of the semiconductor^63–65^. However, it is seen from the band diagram that the material using local functional (GGA) is a direct bandgap semiconductor, which is found at the R point in the Brillouin zone. To observe the individual atomic contribution to the conductivity when external stimuli are applied and to acquire knowledge on band formation, we have also studied the total and partial DOS. The total and partial DOS for the CsSnCl_3_ compound is illustrated in Fig. 2b. It is seen that the valence band, which is very close to the Fermi level (*E*_F_), comprises Cl-3*s* orbital and Sn-5*s*/5*p* orbitals, while the nearest conduction band (very close to the *E*_F_) is composed of Sn-5*p* and Cl-3*s* orbitals. The prime contribution to the conductivity could come from the Sn element, as seen in Fig. 2b. The observed electronic contributions of constituent elements support the utilization of the CsSnCl_3_ compound as a solar absorber.

#### Electron charge density map of CsSnCl_3_ compound

The analysis of charge density distribution can reveal the type of chemical bonding that exists in the halide perovskite, CsSnCl_3_. The mapping image of the electron density difference in the (110) and (100) planes is depicted in Fig. 2c,d. Here, it is seen that maximum charge accumulation occurs around the Cl element for both planes, while its depletion is located around the Sn atom/element. In other words, the overlap of electron clouds between these two elements indicates covalent bonding^63,65^. This charge distribution channel strongly supports the covalent bonding nature that exists between Sn-Cl atoms. The charge distribution surrounding the atoms is observed to be nearly a sphere, which is a sign of ionic bonding and can be compared to previously reported perovskites^66,67^. Additionally, the Mulliken population analysis confirms that the Sn–Cl bond’s population value is positive and bigger than zero (0.37), demonstrating the bond's covalent character. On the other hand, the Sn–Cs and Cs–Cl bonds have a negative population value and are therefore determined to be an antibonding character.

#### Fermi surface topology of CsSsCl_3_ compound

The thermal, electrical, and optical properties of a compound can be described and predicted by its Fermi surface topology. Therefore, we also have studied the Fermi surface of the titled compound, which is presented in Fig. 2e,f. It is found that a spherical shape is located at the center (G-point) as a hole pocket. An open window is noticed at the six faces along the G-X direction for example. The eight open surfaces can be found at eight corners and are considered electron pockets. As a result, there are Fermi surfaces that resemble both electrons and holes, which suggests that the aforementioned material has multiple band features. Finally, it can be concluded that the highly dispersive band comprising the hybridization between Sn-5*p* and Cl-3*s* orbitals could be mainly responsible for the electronic conductivity.

#### Optical properties of CsSnCl_3_ compound

**Figure 3:**
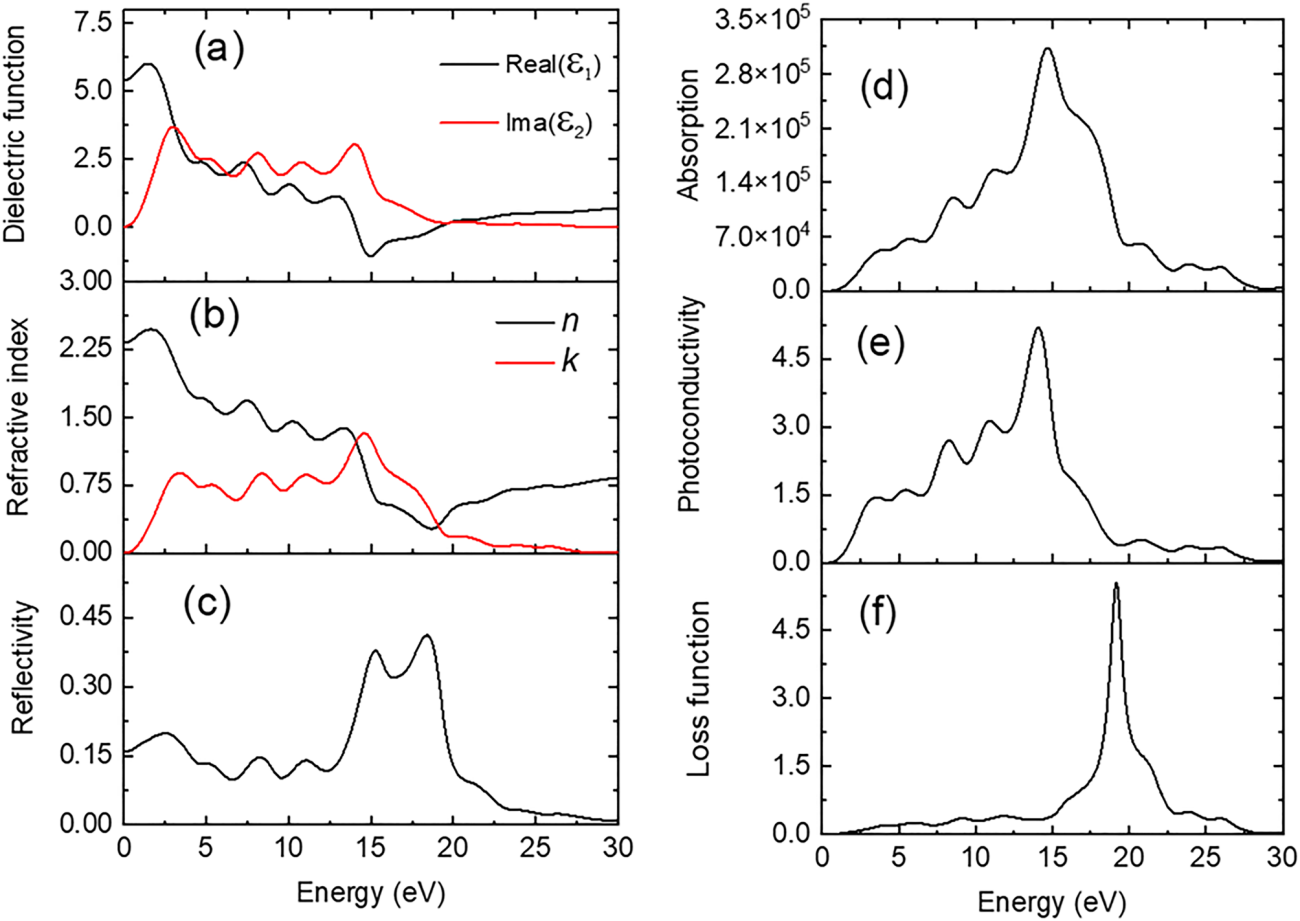
Photon energy dependency of (**a**) dielectric function, (**b**) refractive index and (**c**) reflection coefficient; and (**d**) absorption coefficient, (**e**) photoconductivity and (**f**) loss function for halide perovskite, CsSnCl_3_ along (110) plane.

The photon energy-dependent dielectric function [real part, Ɛ_1_(ω) and imaginary part, Ɛ_2_(ω)] for CsSnCl_3_ has been studied in the energy range from 0 to 30 eV which is depicted in Fig. 3a. The corresponding formulas and theory can be found elsewhere^68^. The static value of the dielectric constant [Ɛ_1_(0)] can be estimated from the Penn model given in Eq. (11) ^68^.

11 $$\varepsilon_{1} \left( 0 \right) = 1 + \left( {\frac{{E_{p} }}{{E_{g} }}} \right)^{2} \left[ {1 - \frac{{E_{g} }}{{4E_{F} }} + \frac{1}{3}\left( {\frac{{E_{g} }}{{4E_{F} }}} \right)^{2} } \right]$$

where *E*_p_, *E*_g_, and *E*_F_ indicate plasma energy, energy bandgap, and Fermi energy, respectively. The value of Ɛ_1_(0) signifies the index of refraction, which is very essential to fabricate many optoelectronic devices. The Ɛ_1_(0) value is noted to be 5.42. The highest value of Ɛ_1_(ω) is found to be ~ 6 at a photon energy of 1.57 eV and then gradually decreases to reach zero as well as negative values at 13.2 and 15 eV, respectively. After that, it is back to zero again at around 19.0 eV. This scenario of negative dielectric function in this frequency range is an indication of the Drude behavior of the compound. The absorption function of light can be expressed by the imaginary part of the energy-dependent dielectric function, Ɛ_2_(ω). The highest peak is noted at 3.6 eV and then a decreased tendency of light absorption is found with the increment of photon energy.

The energy-dependent refractive index (*n*) and extinction coefficient (*k*) are shown in Fig. 3b. It is well known that *n* governs light's speed relative to its free space in a compound, while *k* denotes its attenuation in a solid. The value of *n*(0) for CsSnCl_3_ is 2.26 eV. This value is almost constant (variation ~ 1.6%) in the IR and visible light energy region and then declines as light energy increases as shown in Fig. 3b. The greatest value of the refractive index was 2.28, and we can see that it declines with the increase of radiative light. The value of *k* follows an almost similar nature to that of Ɛ_2_(ω). The refractive index fluctuates with external frequency, indicating that CsSnCl_3_ has photorefractive characteristics.

The energy-dependent reflectance spectrum (*R*) is a crucial optical function for applying Kramers-Kroning relationships to calculate all optical coefficients. The reflectivity spectrum begins with ~ 16% reflectivity in this solar absorber material (Fig. 3c). The R-value (0. 16) at zero frequency is assumed to be the static component of reflectivity. It is seen from Fig. 3c that this spectrum is almost constant (variation ~ 2.9%) in the IR and visible light region (0–3.1 eV) and then broadly decreased in the near UV region owing to the inter-band transitions up to 6.7 eV. After that, some notable peaks are found at 8.43, 11.0, 15.27, and 18.57 eV and then again sharply decrease to reach zero value at ~ 30 eV of photon energy. The maximum reflectivity (0.41) is seen in the infrared range (3.85 eV) for the intra-band transitions in the compound, according to Fig. 3c.

The term absorption coefficient (α) offers crucial information about solar energy conversion efficiency by displaying the number of photons that a substance has absorbed. As shown in Fig. 3d, the absorption spectrum starts with a photon energy of ~ 1 eV, indicating that CsSnCl_3_ has an energy gap between the valence and conduction band (semiconducting nature). The absorption coefficient spectrum was increased with some prominent shoulder peaks at 5.66, 8.52, and 11.29 eV which suggests that the photon absorption initiates in the visible spectrum. The maximum value of the absorption coefficient was recorded at 14.84 eV. It is significant to note that with absorbed light, a semiconductor’s electrical conductivity and, consequently, photoconductivity, increase.

The photoconductivity of a material mainly depends on how much light energy is absorbed into the material. As shown in Fig. 3e, photoconductivity increases linearly in the visible light energy range, and then in the UV region, it rises with some distinct peaks at 3.3, 5.43, 8.36, and 11 eV. The maximum photoconductivity (4.8) is achieved when the incident photon energy is 14 eV. However, the photoconductivity typically decreases with photon energy after reaching its peak value.

The energy loss spectrum (L) of CsSnCl_3_ is depicted in Fig. 3f, which illustrates how much energy a fast electron loses while moving through a molecule. The energy loss of moving carriers is simply explained by the energy loss function. It is seen that energy loss slowly increased up to the light energy of 15.0 eV and then it increased rapidly. The highest energy loss could be obtained at around 19 eV, which is called plasma frequency (*ω*_p_). Because of the higher photon energy than E_g_, it is evident that the majority of energy loss happens in the UV range.

### Analysis of SCAPS-1D results

In this section, the PV performance of the ITO/ETL/CsSnCl_3_/HTL/Au structure is evaluated by keeping carrier concentration, thickness, and defect density of the ETL, absorber, and HTL layers constant along with 800 nm of CsSnCl_3_ absorber thickness, acceptor doping concentration of 10^18^ cm^−3^, and defect densities of 10^15^ cm^−3^ (Tables 2 and 3).

#### Effect of ETL

**Figure 4:**
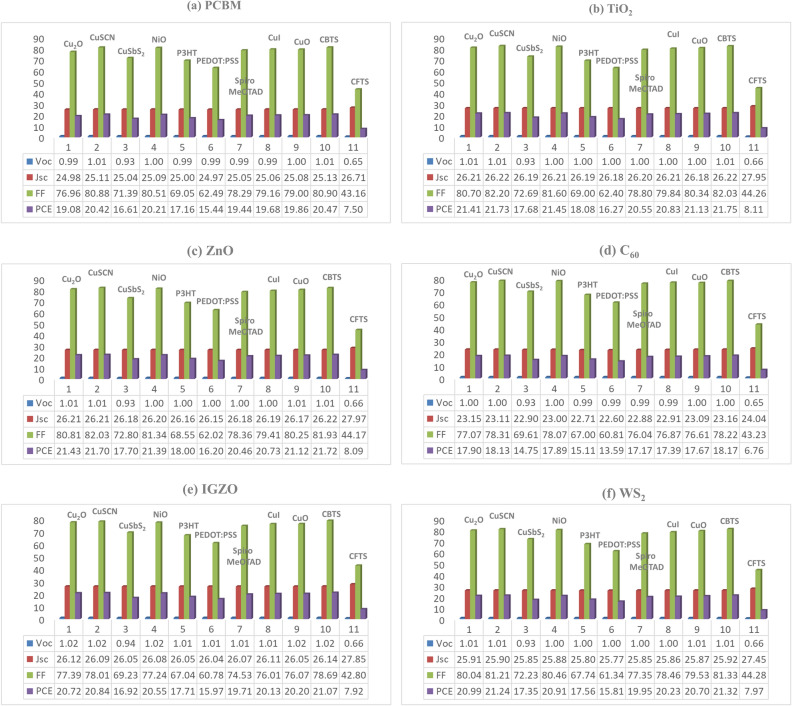
Optimization of CsSnCl_3_ on PSC characteristics, i.e., *V*_OC_ (V), *J*_SC_ (mA/cm^2^), *FF* (%) and *PCE* (%) for various HTLs with Au as back metal contact and ETLs: (**a**) PCBM, (**b**) TiO_2_, (**c**) ZnO, (**d**) C_60_, (**e**) IGZO, and (**f**) WS_2_.

**Figure 5:**
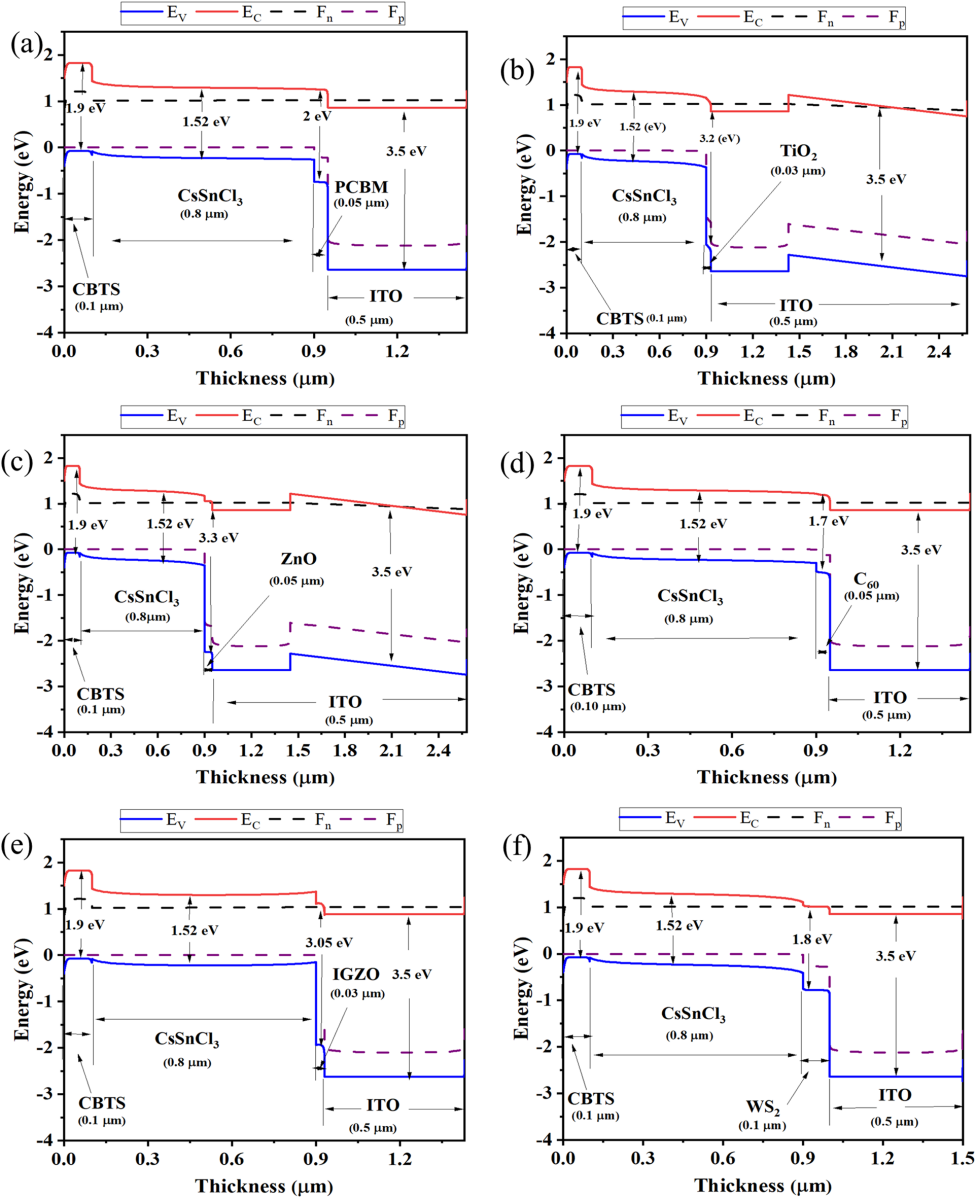
Band diagram of six optimized devices of CsSnCl_3_ with ETLs (**a**) C_60_, (**b**) IGZO, (**c**) PCBM, (**d**) TiO_2_, (**e**) WS_2_, (**f**) ZnO.

**Figure 6:**
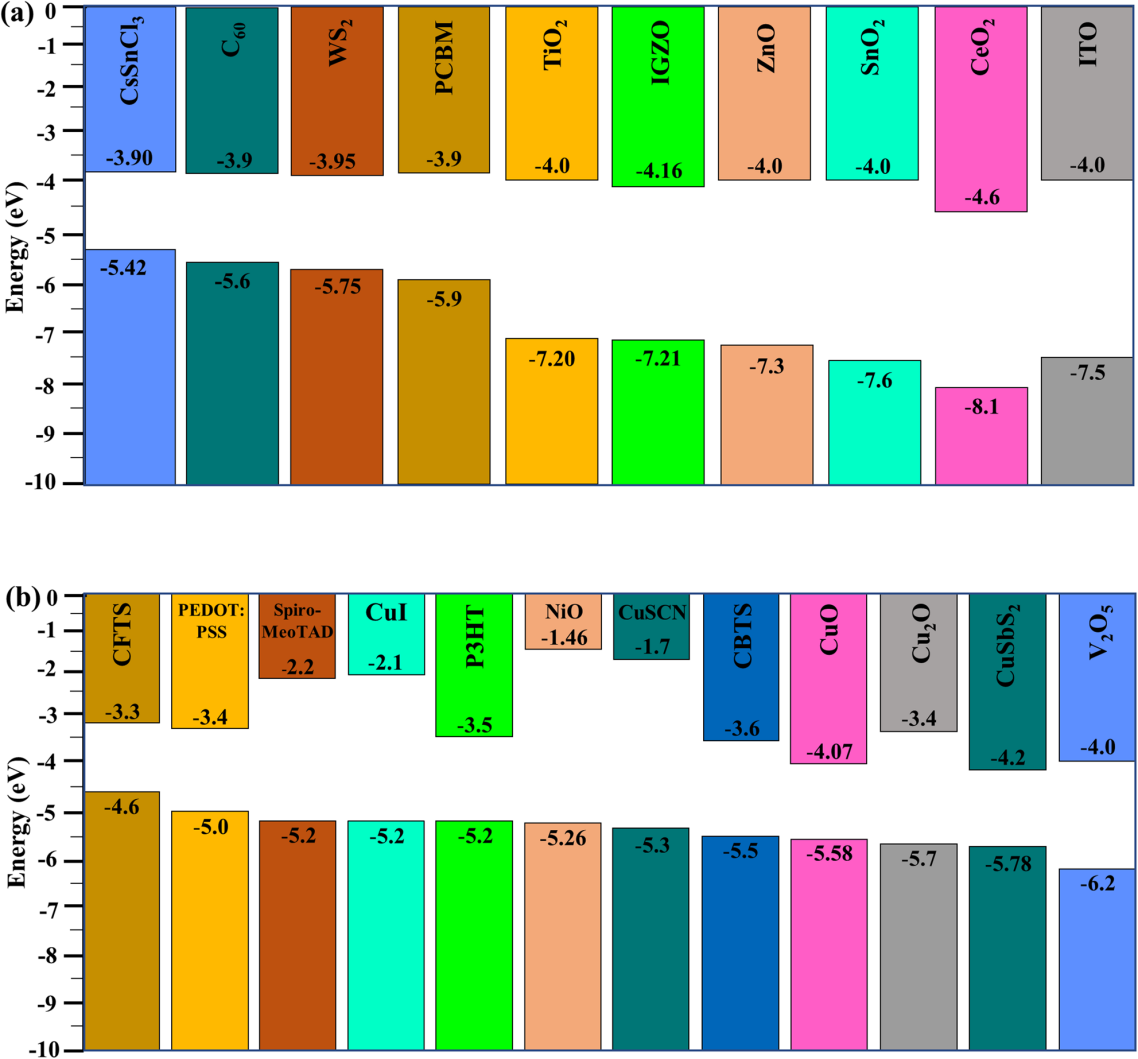
Energy level alignment of the related (**a**) ITO, ETLs, and absorber CsSnCl_3_, and (**b**) HTLs.

Figure 4 depicts that, the ETLs such as TiO_2_, ZnO, IGZO, WS_2_, PCBM, and C_60_ were inserted into the pristine heterostructure to obtain a configuration showing the highest photovoltaic performance. Our findings illustrate that ETLs of TiO_2_ and ZnO with a wide bandgap of 3.2 eV and 3.3 eV respectively and suitable electronic properties offer favorable transparency and band alignment (Figs. 5 and 6), which exhibited *PCE* of around 22% (Table 5), which is consistent with the previous report^69^. In contrast, ETLs like CeO_2_, PCBM, and C_60_ suffering from unsuitable bandgap and/or band alignment showed poor performance relatively. This finding shed light on the unsuitability of these candidates for SC configuration. Further, the optimum ETLs thickness of 30–50 nm, the donor concentration of 10^17^–10^18^ cm^−3^, and defect densities of 10^15^ cm^−3^ were obtained. The highest performance was found with the *PCE* of 21.75%, *J*_SC_ of 26.22 mA/cm^2^, *V*_OC_ of 1.01 V, and *FF* of 82.03% with ITO/TiO_2_/CsSnCl_3_/CBTS/Au heterostructure as shown in Table 5.

**Table 5:** Optimized performance parameters of the best combination for each of the ETLs and CBTS HTL.

Optimized device	Cell thickness (μm)	*V*_oc (V)_	*J*_sc_ (mA/cm^2^)	*FF* (%)	PCE (%)
ITO/PCBM/CsSnCl_3_/CBTS	0.5/0.05/0.8/0.1	1.01	25.13	80.9	20.47
ITO/TiO_2_/CsSnCl_3_/CBTS	0.5/0.03/0.8/0.1	1.01	26.22	82.03	21.75
ITO/ZnO/CsSnCl_3_/CBTS	0.5/0.05/0.8/0.1	1.01	26.22	81.93	21.72
ITO/C_60_/CsSnCl_3_/CBTS	0.5/0.05/0.8/0.1	1.00	23.16	78.22	18.17
ITO/IGZO/CsSnCl_3_/CBTS	0.5/0.03/0.8/0.1	1.02	26.14	78.69	21.07
ITO/CeO_2_/CsSnCl_3_/CBTS	0.5/0.1/0.8/0.1	0.85	26.06	65.37	14.43
ITO/WS_2_/CsSnCl_3_/CBTS	0.5/0.1/0.8/0.1	1.01	25.92	81.33	21.32

#### Effect of HTL

The HTLs such as CBTS, CuSCN, NiO, Cu_2_O, V_2_O_5,_ and CFTS with more than twelve different combinations were studied (all are not shown here). The HTLs of CBTS offered much more favorable band alignment (Figs. 5 and 6), therefore, it exhibited the highest *PCE* around 22% for CsSnCl_3_ absorber-based SCs (Table 5). In contrast, the NiO, Cu_2_O, V_2_O_5,_ and CFTS showed poor performance in comparison to others. So, there has been a dramatic improvement in performance while using the inorganic HTL. Since the HTL is made of inorganic materials that are more stable, transparent, and band-aligned, it has these advantages over organic HTL. Due to its distinct crystalline structure, light absorption, and atomic size, HTL CBTS, an earth-abundant material, performs well in each set of ETL^29,37,38^.

#### Band alignment of CsSnCl_3_-based heterostructure with different ETLs

Figures 5a–f show the band alignment of different CsSnCl_3_ absorber-based heterostructures with quasi-Fermi levels of electron and holes as F_n_ and F_p_ with conduction band minima and valence band maxima of E_C_ and E_V_ respectively. F_p_ is aligned with E_V_ in each type of ETL, whereas F_n_ and E_C_ continue harmonically. For TiO_2_ and ZnO ETLs, the bandgaps are nearly equal in comparison with the rest of the ETLs (Fig. 6), thereby, resulting in an almost equivalent performance with the same heterostructure. On the other hand, the F_p_ and E_V_ remained at the same level for CBTS HTL and F_n_ cross through E_C_, which opposes holes entering from ETLs and electrons from HTL. Moreover, the rear contact Au collects holes from the HTL, whereas the front contact ITO collects electrons effectively. Herein, the gold was considered as the rear contact with a work function (WF) of 5.1 eV and indium dioxide with a WF of 4.0 eV acts as the front contact.

#### J-V and QE characteristics

**Figure 7:**
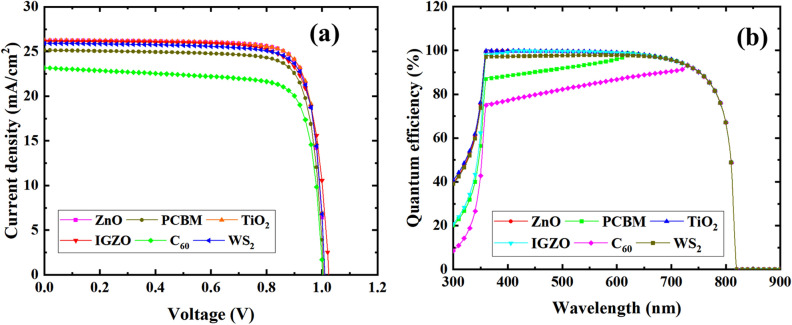
Effect of (**a**) *J–V*, and (**b**) QE for six studied devices.

Figure 7 presents the *J–V* characteristics and *QE* for different ETLs for the device configuration of ITO/ETLs/CsSnCl_3_/CBTS/Au heterostructure. The *QE* computed in this manuscript is actually the external quantum efficiency (*EQE*). The maximum photocurrent was obtained in ITO/TiO_2_/CsSnCl_3_/CBTS/Au PSC while the minimum for ITO/C_60_/CsSnCl_3_/CBTS/Au (Fig. 7a). The favorable band alignment of TiO_2_ (*E*_g_ ~ 3.2 eV) ETL offers a higher current density while lower current in C_60_ (*E*_*g*_ ~ 2 eV) ETL owing to unsatisfactory band alignment. Thus, the ETLs band alignment influences the flow of photogenerated electrons or holes in CsSnCl_3_-based PSCs, which is consistent with previous reports^57,70^.

The corresponding *QE* as a function of wavelength (*λ*) was studied in the range of 300–900 nm as shown in Fig. 7b. This *QE* started to increase from 300 nm and dropped to 820 nm corresponding to the band edge of each active material. The *QE* was found maximum for ITO/TiO_2_/CsSnCl_3_/CBTS/Au heterostructure as expected from like *J–V* characteristics and the minimum for ITO/C_60_/CsSnCl_3_/CBTS/Au configuration. The higher band gap of TiO_2_ allows the higher photon to be absorbed in the CsSnCl_3_ absorber, resulting in the generation of higher current density. On the contrary, C_60_ with a smaller band gap (*E*_g_ ~ 2 eV) result in lower absorption in the absorber, therefore suppressing photocurrent. Thus, the band orientation of the ETLs affects significantly the light absorption, and consequently the photocurrent of cells^71^.

#### Effect of absorber and ETL thickness on cell performance

**Figure 8:**
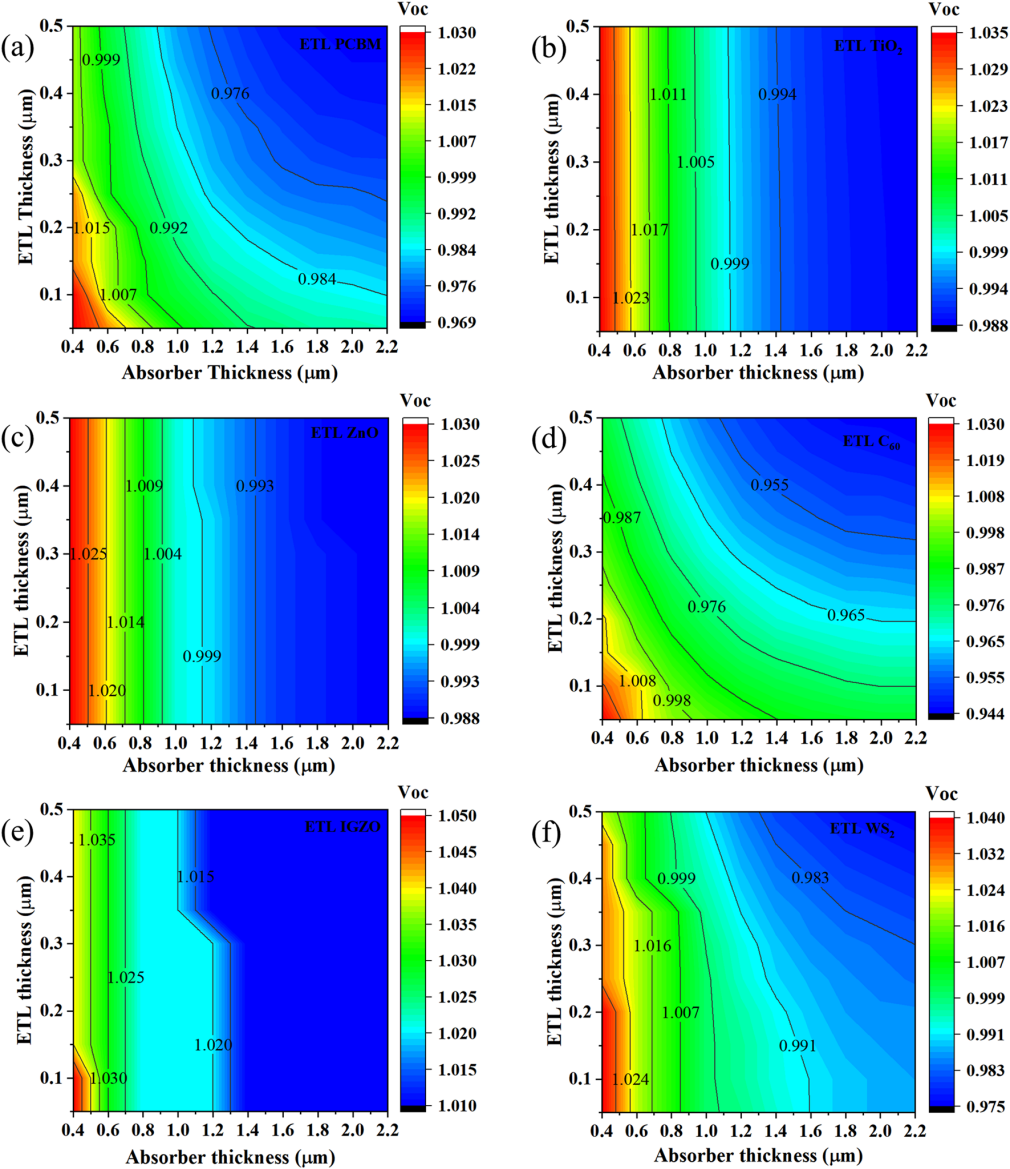
Contour mapping of *V*_OC_ for CsSnCl_3_ absorber and ETLs (**a** C_60_, **b** IGZO, **c** PCBM, **d** TiO_2_, **e** WS_2_, and **f** ZnO) thickness.

**Figure 9:**
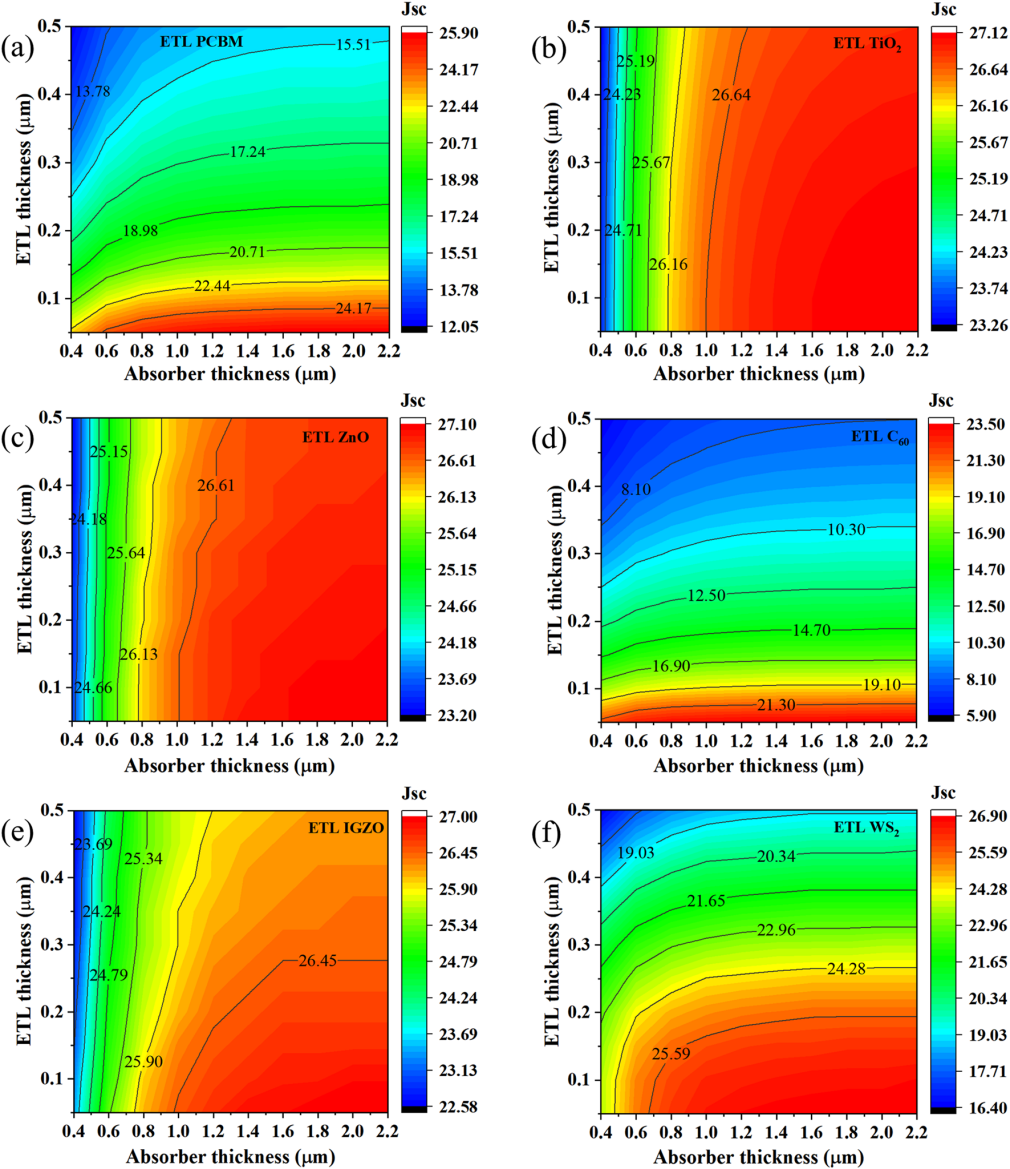
Contour mapping of *J*_SC_ for CsSnCl_3_ absorber thickness and ETLs (**a** C_60_, **b** IGZO, **c** PCBM, **d** TiO_2_, **e** WS_2_, and **f** ZnO) thickness.

**Figure 10:**
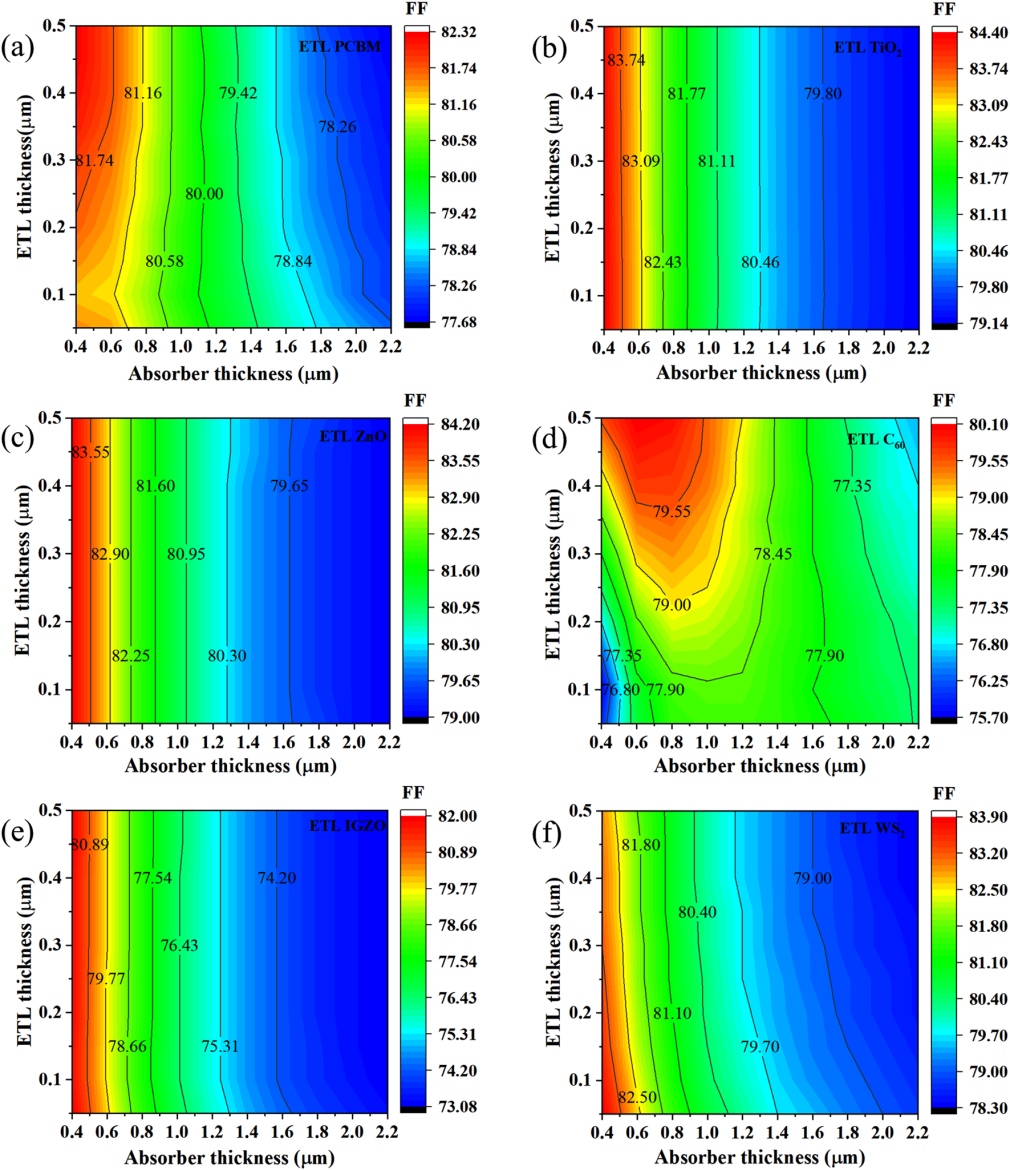
Contour mapping of *FF* for CsSnCl_3_ absorber thickness and ETLs (**a** C_60_, **b** IGZO, **c** PCBM, **d** TiO_2_, **e** WS_2_, and **f** ZnO) thickness.

**Figure 11:**
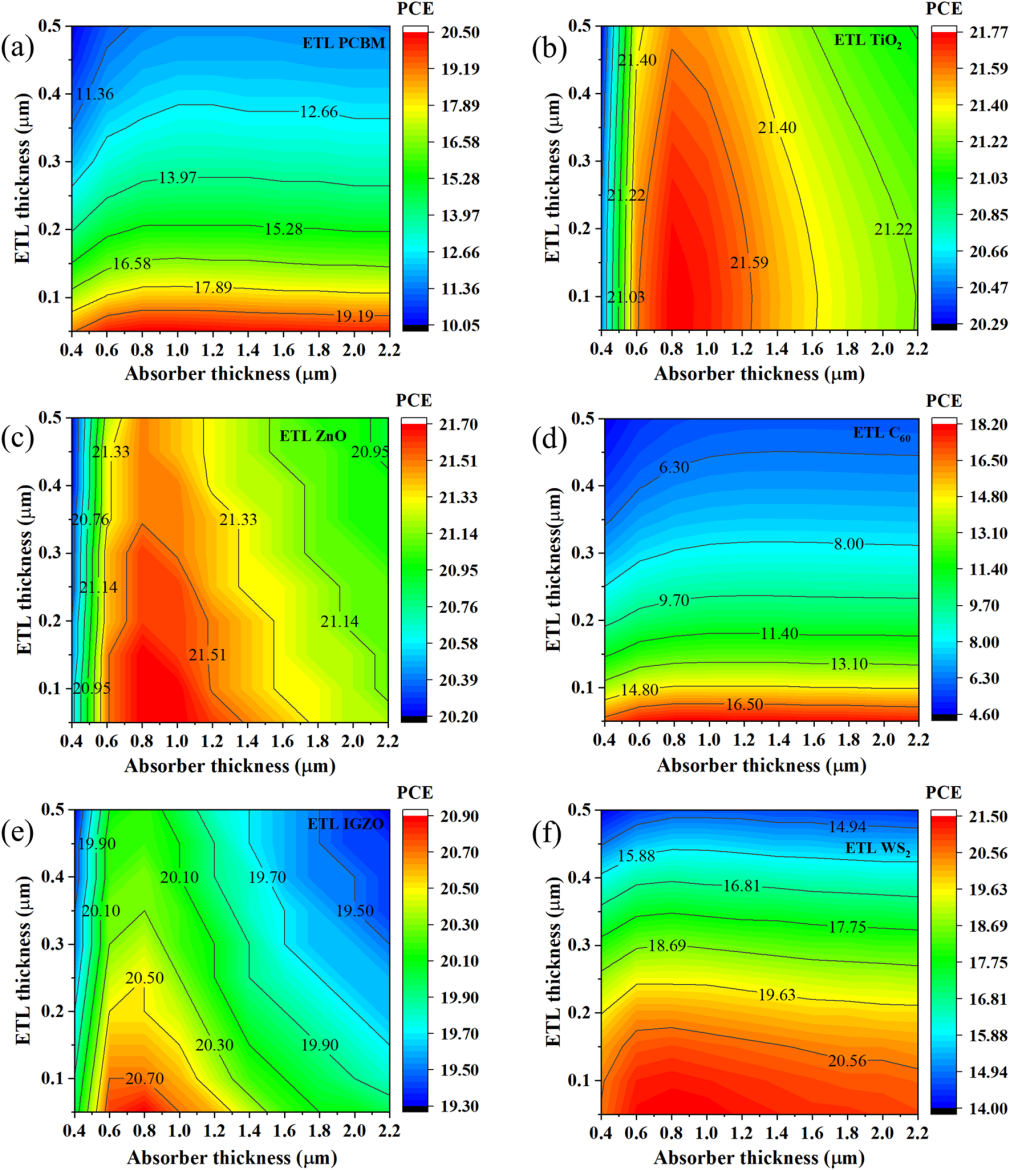
Contour mapping of PCE for CsSnCl_3_ absorber thickness and ETLs (**a** C_60_, **b** IGZO, **c** PCBM, **d** TiO_2_, **e** WS_2_, and **f** ZnO) thickness.

For the CsSnCl_3_-based PSCs, the contour maps of the computed *V*_OC_, *J*_SC_, *FF*, and efficiency with variable ETL thickness (50 nm to 500 nm) and absorber thickness (400 nm to 2200 nm) are depicted in Figs. 8, 9, 10 and 11. *V*_OC_ was maximum at ~ 1.03–1.05 V for each device at an absorber thickness of 400 nm and at a varied ETL thickness from 50 to 100 nm. Among all devices, *V*_OC_ was found to be a maximum of 1.050 V at a 400 nm-thick-CsSnCl_3_ absorber and 50 nm-thick IGZO ETL as depicted in Fig. 8e. And ETLs like PCBM, TiO_2_, and ZnO showed a slightly smaller *V*_OC_ of 1.030 V at an absorber and ETL thickness of 400 nm and 100 nm respectively. Since the built-in potential formed by the six chosen ETLs is nearly equal as defined by their electron affinity value in the range of 3.9–4.1 eV, the *V*_OC_ of each device shows almost the same value as ~ 1.03–1.05 V. The slight difference in terms of *V*_OC_ between devices employing different ETL materials is mainly due to the bandgap difference. However, the *V*_OC_ of all the devices tends to decrease with the increase of both absorber and ETL thickness over 500 and 50 nm respectively as observed in Fig. 8. This fact is due to the increased series resistance and reduced photocurrent.

Figure 9 illustrates the *J*_SC_ for different ETL configurations whereas the highest *J*_SC_ was observed in the range of ~ 23.50–27.12 mA/cm^2^ at an absorber thickness of ≥ 800 nm and ETL thickness of 50–100 nm. The highest *J*_SC_ of 27.12 mA/cm^2^ was obtained by TiO_2_ ETL with absorber thickness of ≥ 1400 nm and ETL thickness of 150 nm for each of the six studied heterostructures. On the contrary, a smaller *J*_SC_ of 23.50 mA/cm^2^ was obtained by C_60_ ETL with an absorber thickness of ≥ 1400 nm and ETL thickness of 50 nm. The relatively higher *J*_SC_ observed in TiO_2_, ZnO, and IGZO ETLs-based heterostructure owing to their wide band gap and good light penetration depth through these materials, resulting in, the allowance of the higher amount of incident light to be absorbed in the absorber layer. In addition, higher absorber thickness confirms the absorption of higher wavelength light spectra, resulting in an improved *J*_SC_. However, the *J*_SC_ of all devices starts to decrease for absorber thickness of ≥ 1000 nm and ETL thickness of ≥ 100 nm because of the domination of recombination at thicker device structure than carrier lifetime.

Figure 10 shows the *FF* for different ETLs configurations, where the highest *FF* was observed in the range of ~ 80.1–84.4% corresponding to the absorber and ETL thickness of 400–600 nm and 400–500 nm respectively for the investigated heterostructures. Maximum *FF* of 84.4% was obtained for TiO_2_ ETL with absorber and ETL thickness of 400 and 450 nm respectively and the *FF* was relatively smaller at 80.1% for C_60_ ETL with the absorber and ETL thickness of 600 nm and 450 nm respectively. So far, the *FF* decreased at an absorber thickness of ≥ 600 nm and ETL thickness of ≤ 400 nm for every heterostructure, owing to the absorber thickness being greater than the diffusion length, thereby generating noticeable recombination of photogenerated charrier in the quasi-neutral region. Besides, reduced ETL thickness will increase the film resistivity which hinders the *FF*. Otherwise, a strong electric field developed across the absorber layer originates from the *FF*, and an increasing absorber thickness causes a weakening of the overall electric field’s strength and thereby formation of a quasi-neutral region in the absorber layer. Besides, the formation of a quasi-neutral layer causes the carriers to transport through diffusion rather than drift, which led to a reduction of *FF* with increasing series resistance.

As a result, the highest efficiency in the range of ~ 18.20–21.77% was obtained for all studied heterostructures at an ETL thickness of 50–100 nm, and absorber thickness of 800–1200 nm (Fig. 11). As expected from the aforementioned *V*_OC_*, J*_SC_ and *FF* results, the efficiency of 21.77% was the highest for TiO_2_ ETL with the absorber and ETL thickness of 800 and 50 nm accordingly. The efficiency of ETL C_60_ was found relatively smaller at 18.20% with absorber and ETL thickness of 1200 and 50 nm respectively. Therefore, the optimum absorber and ETL thickness of 800–1200 and 50–100 nm was obtained for chosen six different ETL configurations. However, the ETLs of TiO_2_, ZnO, and IGZO-with CsSnCl_3_ absorber-based heterostructures were found much more efficient and promising than the rest of the structures. These simulated results are consistent with the previous report^72^.

The effect of ETL thickness variation on the PV parameters, i.e., *PCE*, *FF*, *J*_SC_, and *V*_OC_ is shown in Fig. S1. It is clearly seen that the increase in the thickness of the ETL leads to the degradation in the PV parameters for most of the ETLs, thereby leading to a decrement in *PCE*. This is due to the inefficient transport of charge carriers to the electrodes, the increase in series resistance that degrades the *FF*, and the increase in the probability of recombination with increasing ETL thickness^73^.

#### Effect of series resistance

There are three factors that contribute to series resistance in solar cells: first, the flow of current via the SC’s ETL/PVK and PVK/HTL interfaces (here PVK means “Perovskite”); second, the metal contact/ITO interface resistance; and third, the resistance of the top and rear metal contacts. Series resistance mostly affects the FF reduction, while extremely high values may also have a negative effect on short-circuit current.

Several experimental procedures may be used to control or minimize series resistance. For instance, an ETL film created by thermal oxidation is extremely dense and suppresses recombination at the ETL/PVK interface, thereby lowering series resistance^74^. The ETL film made by thermal oxidation has a thinner ideal thickness compared to the one made by spin-coating, which lowers the series resistance. Additionally, compared to two-step sintering, one-step sintering results in reduced dark current densities, reduced series resistance, and stronger recombination resistance^75^. Additionally, perovskite films were molecularly doped to increase their conductivity and electronic contact with the conductive substrate, which decreased their series resistance^76^.

**Figure 12:**
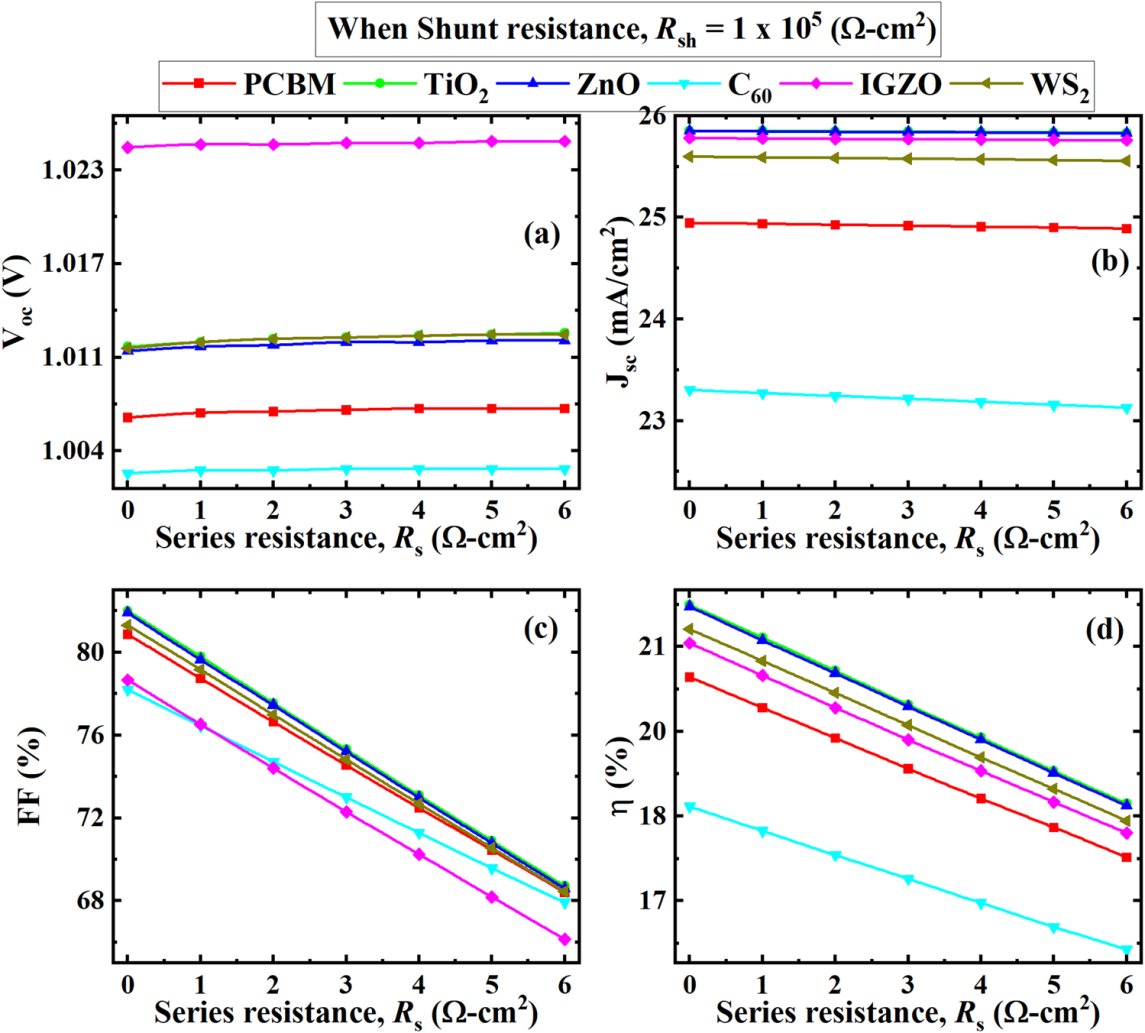
Effect of *R*_S_ on (**a**) *V*_OC_; (**b**) *J*_SC_; (**c**) *FF*; (**d**) *PCE* at an *R*_SH_ = 10^5^ Ω cm^2^.

Figure 12 illustrates the impact of series resistance (*R*_S_) on the PSC performance in the ranges of 0 to 6 Ω cm^2^, at a constant shunt resistance (*R*_SH_) of 10^5^ Ω cm^2^. The *FF* was found as the most affected parameter for the varied *R*_S_ than *V*_OC_, and *J*_SC_.

In Fig. 12, the *FF* decreased drastically from 84.5 to 66% with an increase in *R*_*S*_ from 0 to 6 Ω cm^2^ while the *V*_OC_ and *J*_SC_ were affected insignificantly in every device. The IGZO/CsSnCl_3_/CBTS and TiO_2_/CsSnCl_3_/CBTS PSC structures showed the highest *V*_OC_ and *J*_SC_ respectively whereas C_60_/CsSnCl_3_/CBTS PSC structure showed relatively poor performance. Higher *R*_S_ affects *FF* significantly, thereby the conversion efficiency in the PSCs with different heterostructures. Equations (12) and (13) illustrate the effect of *R*_S_ on solar cell parameters, especially short circuit current *I*_SC_.

12 $$I_{SC} = I_{0} \left( {e^{{qV_{OC} /nKT}} - 1} \right)$$

13 $$I_{SC} = I_{L} - I_{0} \left( {e^{{qV_{OC} /nKT}} - 1} \right) - \frac{{V_{OC} + I{\text{scrs}}}}{rsh}$$

where *I*_L_ denotes light-induced current and $rsh$ denotes shunt resistance. It is obvious from the formula above as *R*_S_ increases, the *I*_SC_ value drops. The decrease in efficiency and FF is primarily due to this^77,78^.

#### Effect of shunt resistance

Leakage current and non-geminated recombination losses are responsible for the considerable power losses brought on by the existence of a shunt resistance in PSCs. Due to the creation of pinholes and the metal filling of these pinholes extending to the junctions, there are occasional partial shorts of the junctions in practical solar cells. Low shunt resistance results in power losses in solar cells by giving the current produced by light an alternative path. A similar diversion lowers the voltage generated by the solar cell and lowers the current passing through the junction of the SC. Since there would be a lower light-generated current at low light levels, the influence of shunt resistance is more severe. Accordingly, the effect of losing this current to the shunt is higher. Additionally, the influence of resistance in parallel is significant at lower voltages when the effective resistance of the SC is considerable. Various techniques are used to control or reduce shunt resistance. For instance, a simple way for improving SnO_2_’s electron transport layer (ETL) involves doping the precursor nanoparticles with modest quantities of a Pb source, which effectively raises the shunt resistance. So, adjusting the Pb quantity makes it simple to alter the *R*_sh_ value^79^.

**Figure 13:**
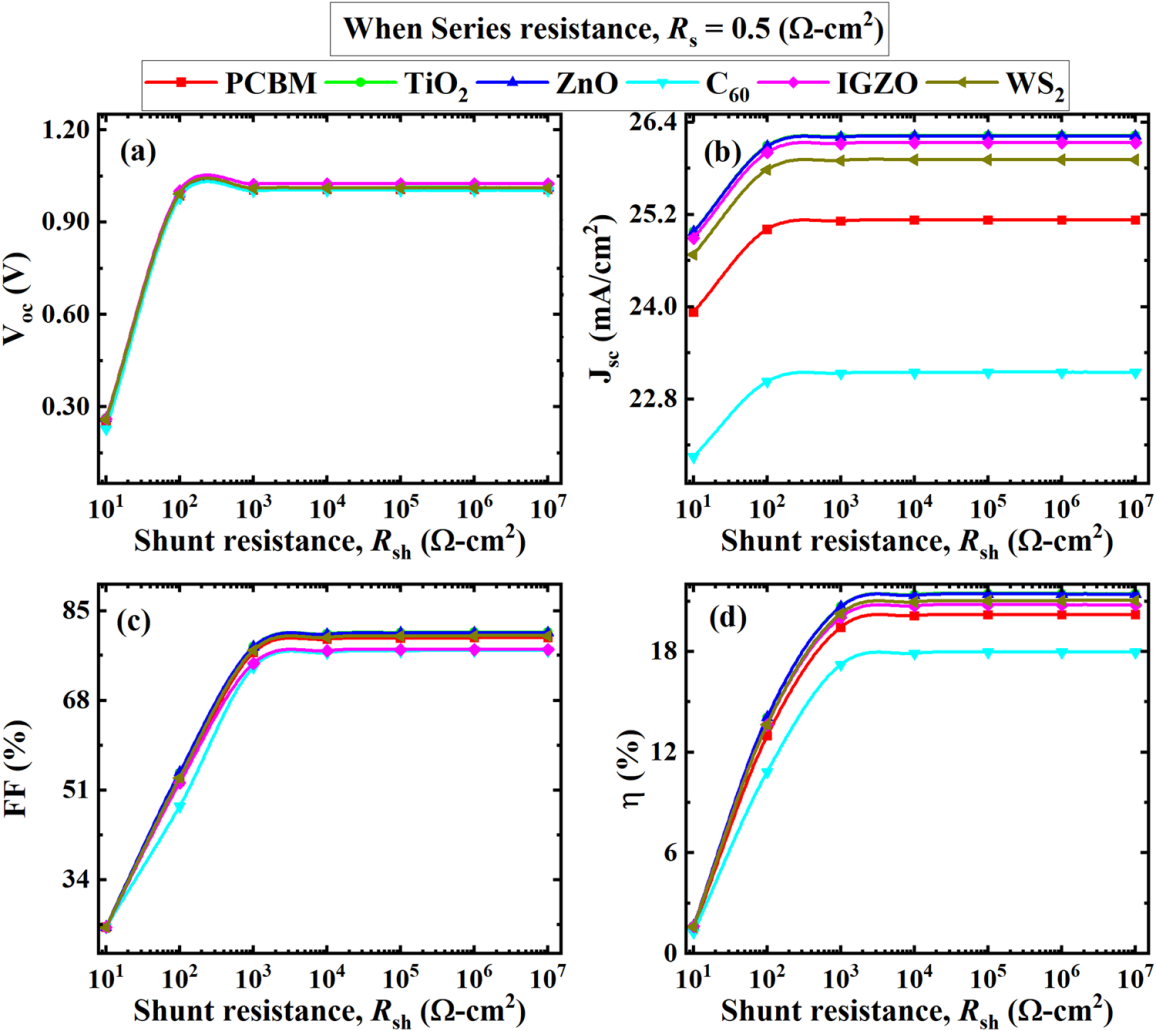
Effect of *R*_SH_ on (**a**) *V*_OC_; (**b**) *J*_SC_; (**c**) *FF*; (**d**) *PCE* at an *R*_*S*_ = 0.5 Ω cm^2^.

Figure 13 shows the effect of *R*_SH_ in the ranges of 10–10^7^ Ω cm^2^ with ITO/ETL/CsSnCl_3_/CBTS/Au heterostructure at a constant *R*_S_ of 0.5 Ω cm^2^. The figure illustrates the significant variation of PV parameters *V*_OC_, *J*_SC_, *FF*, and *PCE* at a varied shunt resistance. Herein, the PV parameters of *V*_OC_, *J*_SC_, *FF*, and consequently *PCE* increase markedly from 2 to ~ 23% when *R*_*SH*_ increases from 10 to 10^3^ Ω cm^2^. The *PCE* reached the highest value of ~ 22% at *R*_SH_ of 10^4^ and remained constant up to 10^7^ Ω cm^2^ or beyond *R*_SH_. The primary origin of *R*_SH_ is the defects formed during the manufacturing process. The structure becomes a low-resistance path for current flow at a higher *R*_SH_ value^56,80^. Herein, the *V*_OC_ increased with shunt resistance up to 8000 Ω cm^2^ while the *J*_SC_ was found almost constant as observed in the previous investigation^81^. Thus, a high value of ${R}_{SH}$ of ≥ 10^4^ is favorable to obtain the highest PCE in ITO/ETL/CsSnCl_3_/CBTS/Au PSCs.

##### Effect of temperature

**Figure 14:**
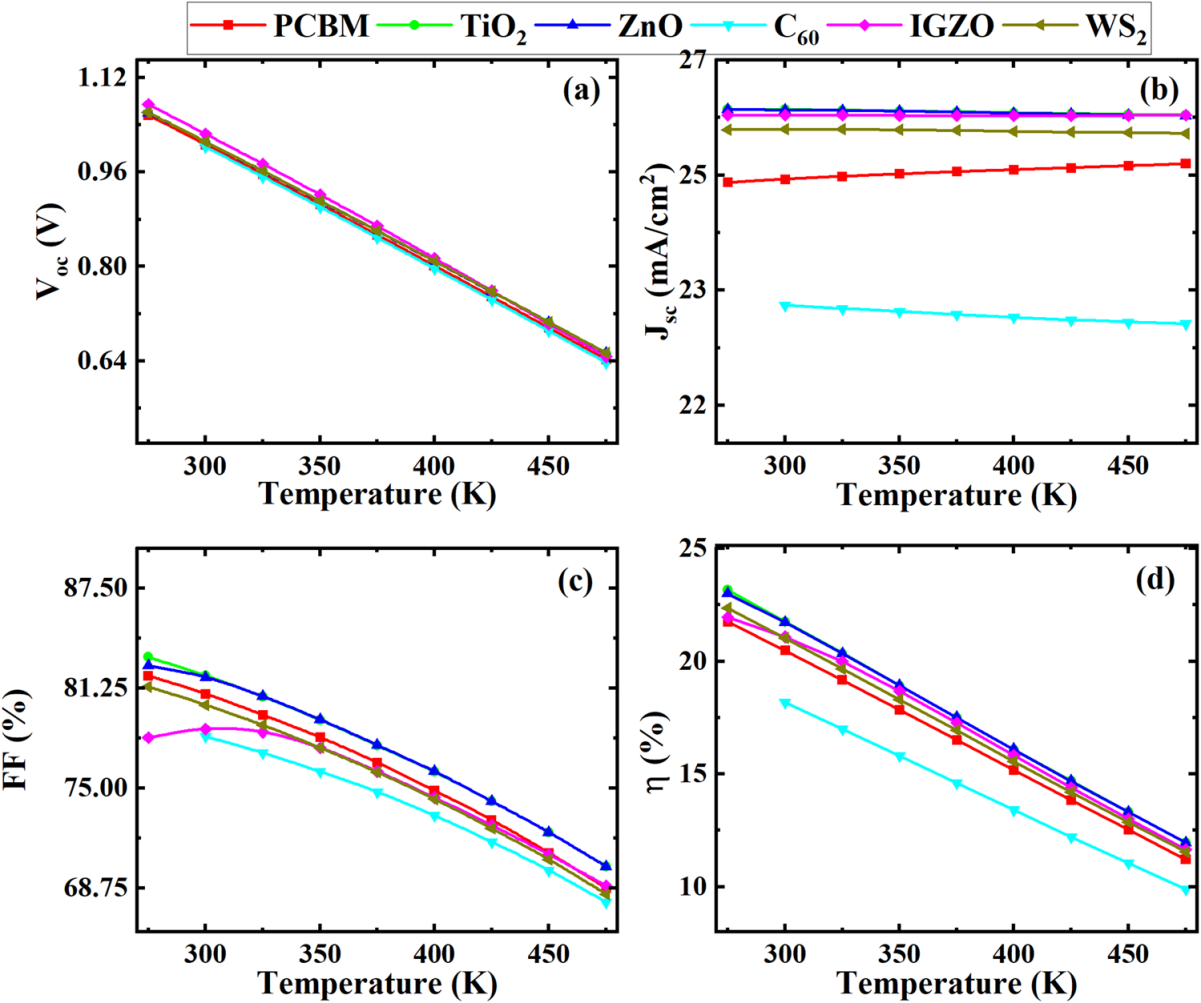
Effect of the variation in temperature from 275 to 475 K on (**a**) *V*_OC_; (**b**) *J*_SC_; (**c**) *FF*; and (**d**) *PCE*.

Figure 14 shows the impact of temperature changes on the performance benchmarks for the six devices, including *V*_OC_, *J*_SC_, *FF*, and *PCE* corresponding to working temperature from 275 to 475 K for an ITO/ETL/CsSnCl_3_/CBTS/Au heterojunction PSC under 1000 Wm^−2^ solar light.

For classical semiconductors, the relationship between temperature and solar cell characteristics is well described^82^. From Fig. 14, the characteristic mainly affected by temperature rise in traditional solar cells is *V*_OC_, which falls with temperature increase, owing to a continuous increase in the saturation current with temperature^83^. The shift in intrinsic carrier concentrations that occurs when the temperature rises cause higher rates of recombination, which in turn has a major impact on the saturation current^83^.

The reliance of *J*_SC_ for PSCs may be roughly described as a linear connection, the same as how it is for *V*_OC_^84^. The *J*_SC_ in an SC is often defined as the product of the ideal current and the collecting fraction. The ideal current is the current that might be created if most incident photons with energies greater than the bandgap were absorbed without losses, whereas the collection fraction is a consequence of charge carrier reflection, transmission, parasitic absorption, and recombination in the SC. From Fig. 14, the variations in *J*_SC_ with temperature are significantly lower than the changes in *V*_OC_. Notwithstanding the commonly approved linear dependence of *J*_SC_ on the temperature in conventional SCs, it is unclear how the *J*_SC_ shifts with temperature in PSCs because increasing the bandgap causes a decrease in the ideal current while the collection fraction rises typically with temperature^85^. As a result, depending on which of these factors predominate, the *J*_SC_ may drop or rise with temperature.

#### Effect of capacitance and Mott-Schottky

**Figure 15:**
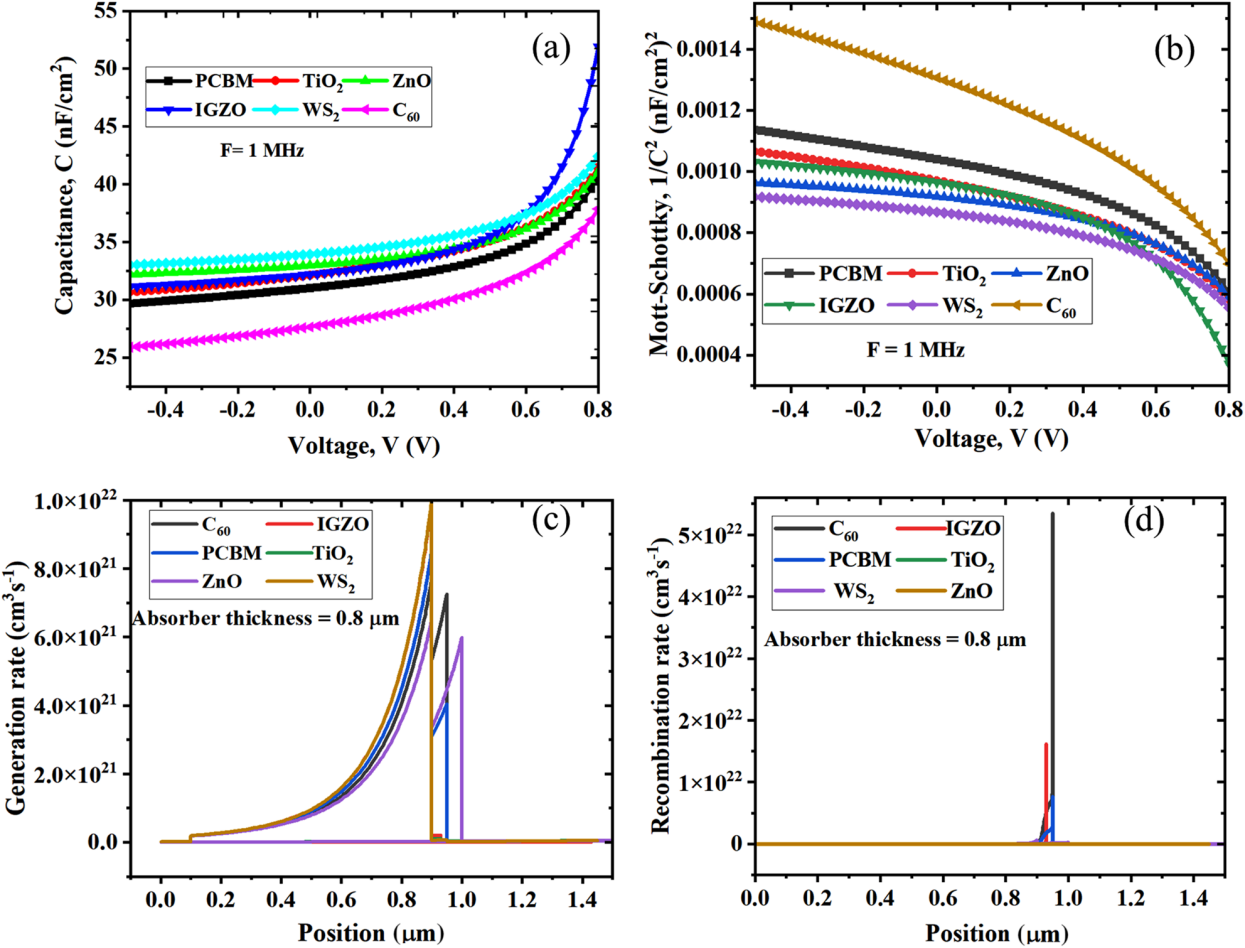
(**a**) The capacitance–voltage (C–V) response, (**b**) Mott-Schottky (1/C^2^) response, (**c**) generation rate, (**d**) recombination rate for absorber CsSnCl_3_-based heterostructures with six different ETLs.

The influence of capacitance and Mott-Schottky versus a voltage range of − 0.5 V to 0.8 V with a fixed frequency of 1 MHz for six different configurations of CsSnCl_3_-based PSCs with different ETLs, is shown in Fig. 15a,b respectively. In Fig. 15a, the capacitance increases exponentially with an increase in supply voltage and reaches saturation. The highest amount of capacitance of 52.5 C was observed for IGZO ETL-based device, whereas the lowest capacitance for C_60_ ETL at 0.8 V. At zero bias, the device is in depletion condition; however, when a forward bias of around 0.5 V is applied, the depletion width falls to a value that is almost equal to the thickness of the absorber layer. As a result, the capacitance rises for further applied forward bias voltage retaining a consistent Mott-Schottky relationship. The current is only allowed to surpass the contact's saturation current during voltage spikes, contrary to what has previously been seen, where it is considerably reduced during low voltages.

The built-in potential (*V*_*bi*_), which distinguishes between the activities of electrode operation and doping level, can be calculated with the help of the widely used Mott-Schottky (MS) method. The intercept point on the x-axis of the C–V curve typically corresponds to the *V*_*bi*_ of the respective junction and the intercept point of the 1/C^2^–V curve denotes the concentration of occupied trapping centers. With an increase in *V*_*bi*_ values, the MS values fall for a particular device. Herein, the simulated results obtained at an identical condition of all of the significant criteria for each of the devices were comparable and consistent with earlier reports^70^.

#### Effect of generation and recombination rate

The carrier generation and recombination rates for CsSnCl_3_-based PSCs are shown in Fig. 15c,d at a range of 0.0–1.5 μm. When an electron is stimulated from the valence band to the conduction band due to the absorption of a photon, a hole is created in the valence band, and this process produces electron–hole pairs. According to the findings, all the device generation rates peak between 0.9 and 1.0 μm. SCAPS-1D calculates the formation of electron–hole pairs G(x) using the arriving photon flux N_phot_ (λ, x), and Eq. (14) analyses this photon flux to show the value of G(x) for every spectrum and location.

14 $$G\left( {\lambda , x} \right) = \alpha \left( {\lambda , x} \right).N_{phot} \left( {\lambda , x} \right)$$

On the other hand, the recombination rate is exactly opposite to the generation process, which unites and eliminates the generated electrons and holes. The rate of recombination in PSCs is influenced by the charge carrier's lifetime and density. The decrease in electron–hole recombination is caused by the defect states that exist within the absorber layer. The maximum recombination rate was observed between 0.9 and 1.0 μm for all studied devices while C_60_ ETL contained the highest recombination peak. The energy levels generated mid-level of the valence-conduction band cause electron–hole recombination within the devices noticeably. The PSCs’ recombination rate distribution can be non-uniform due to grain boundaries and device manufacturing flaws^56,80^.

### Comparison with wxAMPS results and previous work

#### Comparison between SCAPS-1D and wxAMPS results

The simulations were further conducted by the wxAMPS (version 2.0) program to validate the obtained results (Table 6) by SCAPS-1D at a working temperature of 300 K and using an AM1.5G solar spectrum. Both software programs ran simulations using absorber thickness, absorber acceptor concentrations, and defect concentrations of 800 nm, 10^15^ cm^-3^, and 10^15^ cm^−3^, respectively to determine how the PV properties of all the CsSnCl_3_ devices are affected. Table 6 presents a comparison of software simulations using SCAPS-1D and wxAMPS. The closeness between the two simulation results (especially *FF* and *V*_oc_) obtained by wxAMPS and SCAPS-1D revealed the validation of obtained results, which are also inconsistent with earlier studies^56,80^.

**Table 6:** Comparison between SCAPS-1D and wxAMPS software simulation results for CsSnCl_3_ PSCs.

Device structure	Software	*Voc* (V)	*Jsc* (mA/cm^2^)	*FF* (%)	*PCE* (%)
ITO/PCBM/CsSnCl_3_/CBTS/Au	SCAPS-1D	1.01	25.13	80.90	20.47
	wxAMPS	0.99	16.79	80.92	13.52
ITO/TiO_2_/CsSnCl_3_/CBTS/Au	SCAPS-1D	1.01	26.22	82.03	21.75
	wxAMPS	1.01	21.25	82.23	17.68
ITO/ZnO/CsSnCl_3_/CBTS/Au	SCAPS-1D	1.01	26.22	81.93	21.72
	wxAMPS	1.00	20.26	80.83	16.46
ITO/C_60_/CsSnCl_3_/CBTS/Au	SCAPS-1D	1.00	23.16	78.22	18.17
	wxAMPS	0.99	17.15	80.94	13.84
ITO/IGZO/CsSnCl_3_/CBTS/Au	SCAPS-1D	1.02	26.14	78.69	21.07
	wxAMPS	1.02	21.51	79.84	17.47
ITO/WS_2_/CsSnCl_3_/CBTS/Au	SCAPS-1D	1.01	25.92	81.33	21.32
	wxAMPS	1.01	21.02	80.93	17.13

#### Comparison of SCAPS-1D results with previous work

Table 7 compares our obtained outcomes to recent experimental and theoretical findings of CsSnCl_3_-based PSCs for a different configuration. The highest experimental *PCE* of 17.93% is reported FTO/PCBM/CsSnCl_3_/PTAA/Au heterostructure. To date, the theoretical research conducted for improving the performance of the CsSnCl_3_ absorber, and the highest *PCE* of < 20.0% was found through simulation. Herein, we have reported the maximum *PCE* of ~ 22.0% for the very first time. Further, we conducted an extensive simulation for finding an effective ETL, HTL, back metal contact, and so on. However, we conducted all of these simulations to identify the ideal pairings for a stellar performance. In addition, experimental work is in demand for the time to validate the theoretical study that will conduct in near future.

**Table 7:** The comparison of PV parameters of CsSnCl_3_-based solar cells.

Device structure	Absorber thickness (μm)	*V*_*oc*_ (V)	*J*_*sc*_ (mA/cm^2^)	*FF* (%)	*PCE* (%)	Ref
FTO/PCBM/CsSnCl_3_/PTAA/Au	1.0	1.30	15.34	89.90	17.93	^57^
ITO/PCBM/CsSnCl_3_/CBTS/Au	0.8	1.01	25.13	80.9	20.47	*
ITO/TiO_2_/CsSnCl_3_/CBTS/Au	0.8	1.01	26.22	82.03	21.75	*
ITO/ZnO/CsSnCl_3_/CBTS/Au	0.8	1.01	26.22	81.93	21.72	*
ITO/C_60_/CsSnCl_3_/CBTS/Au	0.8	1.00	23.16	78.22	18.17	*
ITO/IGZO/CsSnCl_3_/CBTS/Au	0.8	1.02	26.14	78.69	21.07	*
ITO/WS_2_/CsSnCl_3_/CBTS/Au	0.8	1.01	25.92	81.33	21.32	*

*This work.

## Conclusion

A detailed numerical study on CsSnCl_3_ absorber-based Pb-free SCs from 96 configurations has been performed using the SCAPS-1D simulator. Utilizing the most efficient six device configurations among 96 heterostructures, we further investigated the effects of the CsSnCl_3_ and ETL thickness, series and shunt resistance, and operating temperature. Further, the effects of C–V characteristics like capacitance, and Mott-Schottky, along with generation and recombination rates, *J–V* characteristics, and quantum efficiency were also assessed. The TiO_2_ ETL and CBTS HTL-based heterojunction with ITO/TiO_2_/CsSnCl_3_/CBTS/Au device configuration showed the highest PCE of 21.75% with *V*_OC_ of 1.01 V, *J*_SC_ of 26.22 mA/cm^2^, and *FF* of 82.03% from six optimized devices. Meanwhile, the ZnO, WS_2_, IGZO, PCBM, and C_60_-based devices showed *PCE* of 21.72, 21.32, 21.07, 20.47, and 18.17% respectively. Furthermore, these simulated results obtained by SCAPS-1D were reproduced and validated using wxAMPS numerical study. Moreover, first-principles DFT simulations with the CASTEP program were performed to investigate the structural, electrical, and optical characteristics of the CsSnCl_3_ absorber. The calculated band gap for the CsSnCl_3_ absorber was 1.0 eV and the Sn-5*s*/5*p* orbital electrons displayed significant hybridization based on the estimated partial DOS. The charge density difference analysis strongly supports the covalent bonding nature between Sn-Cl atoms. The observed Fermi surface exhibits hole and electron-like pockets, demonstrating the multiband character of the CsSnCl_3_ perovskite. Thus, these extensive simulations with validation results revealed the high potential of CsSnCl_3_ absorber with TiO_2_, ZnO, IGZO, WS_2_, PCBM, and C_60_ ETLs and CBTS HTL, thereby paving a constructive research avenue for the photovoltaic industry to fabricate cost-effective, high-efficiency, and lead-free CsSnCl_3_-based solar cells. Our future perspective is to use combined DFT-SCAPS-Machine Learning technique to critically investigate nontoxic perovskite devices (ABX_3_: A = Cs; B = Sn, Bi, Ge, Ag, and Sb and X = I, Br) employing competitive charge transport materials, thereby offering researchers a clear guidance towards enhancing PCE values.

## Supplementary Information

Supplementary Information.

## Data Availability

The datasets used and/or analysed during the current study available from the corresponding author on reasonable request.
