# Supplementary material for: An extensive study on multiple ETL and HTL layers to design and simulation of high-performance lead-free CsSnCl3-based perovskite solar cells
Source: Sci Rep. 2023 Feb 13;13:2521. doi: 10.1038/s41598-023-28506-2 (PMC9925818; doi:10.1038/s41598-023-28506-2)
Supplement: Supplementary file 1 — Supplementary Information. [file 41598_2023_28506_MOESM1_ESM.pdf]

SUPPORTING INFORMATION

An extensive study on multiple ETL and HTL layers to design and simulation of a high-performance lead-free $CsSnCl_3$-based perovskite solar cells

M. Khalid Hossain[1,*], G. F. Ishraque Toki[2], Abdul Kuddus[3], M. H. K. Rubel[4,**], M. M. Hossain[5], H. Bencherif[6], Md. Ferdous Rahman[7], Md. Rasidul Islam[8], Muhammad Mushtaq[9]

[1]*Institute of Electronics, Atomic Energy Research Establishment, Bangladesh Atomic Energy Commission, Dhaka 1349, Bangladesh*
[2]*College of Materials Science and Engineering, Donghua University, Shanghai 201620, China*
[3]*Graduate School of Science and Engineering, Saitama University, Saitama, 338-8570, Japan*
[4]*Department of Materials Science and Engineering, University of Rajshahi, Rajshahi 6205, Bangladesh*
[5]*Department of Physics, Chittagong University of Engineering and Technology, Chittagong 4349, Bangladesh*
[6]*Higher National School of Renewable Energies, Environment and Sustainable Development, Batna 05078, Algeria*
[7]*Department of Electrical and Electronic Engineering, Begum Rokeya University, Rangpur 5400, Bangladesh*
[8]*Department of Electrical and Electronic Engineering, Bangamata Sheikh Fojilatunnesa Mujib Science & Technology University, Jamalpur 2012, Bangladesh*
[9]*Department of Physics, University of Poonch Rawalakot, 12350, Pakistan*

**Correspondence to:* *khalid.baec@gmail.com; khalid@kyudai.jp; **mhk_mse@ru.ac.bd

Table of content

Figure S1. Effect of ETLs' thickness variation on the (a) *PCE*, (b) *FF*, (c) J_{SC}, and (d) *Voc*. **S2**

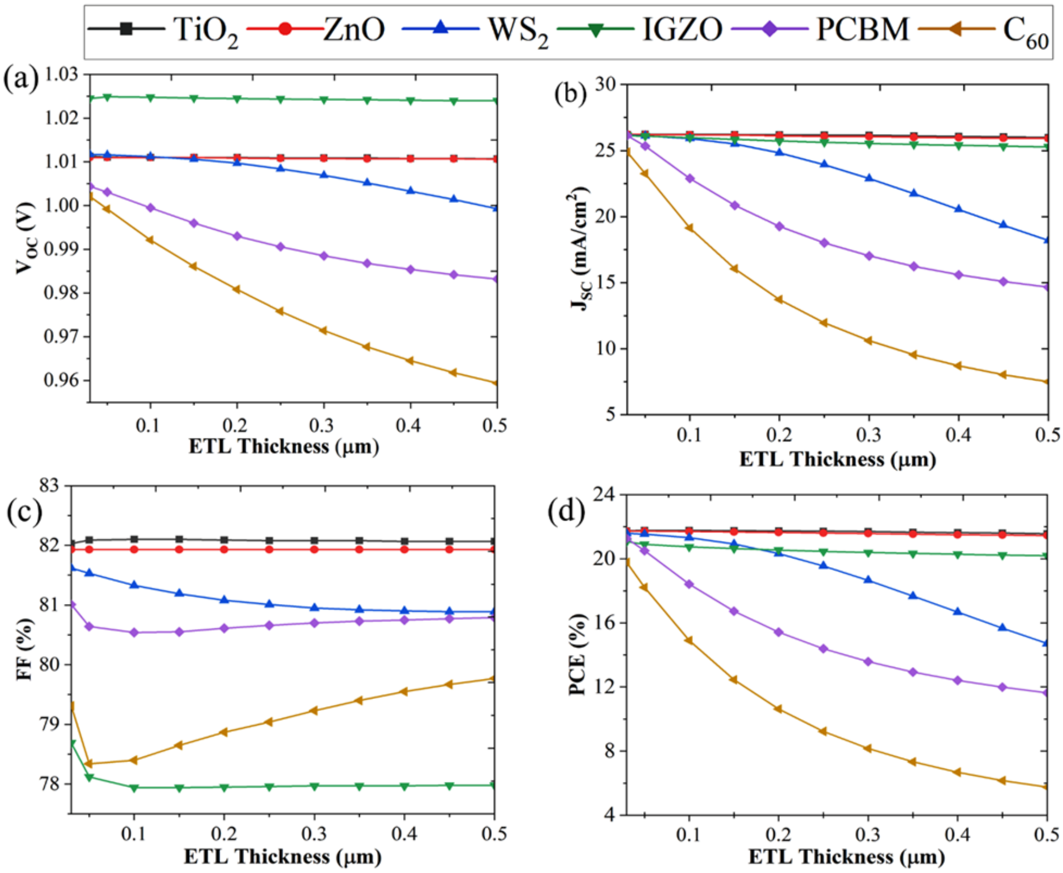

Figure S1: Effect of ETLs' thickness variation on the (a) *PCE*, (b) *FF*, (c) J_{SC}, and (d) *Voc*.
